# Invariance of visual operations at the level of receptive fields

**DOI:** 10.1371/journal.pone.0066990

**Published:** 2013-07-19

**Authors:** Tony Lindeberg

**Affiliations:** Department of Computational Biology, School of Computer Science and Communication, KTH Royal Institute of Technology, Stockholm, Sweden; CSIC-Univ Miguel Hernandez, Spain

## Abstract

The brain is able to maintain a stable perception although the visual stimuli vary substantially on the retina due to geometric transformations and lighting variations in the environment. This paper presents a theory for achieving basic invariance properties already at the level of receptive fields. Specifically, the presented framework comprises (i) local *scaling transformations* caused by objects of different size and at different distances to the observer, (ii) locally *linearized image deformations* caused by variations in the viewing direction in relation to the object, (iii) locally *linearized relative motions* between the object and the observer and (iv) local *multiplicative intensity transformations* caused by illumination variations. The receptive field model can be derived *by necessity* from symmetry properties of the environment and leads to predictions about receptive field profiles in good agreement with receptive field profiles measured by cell recordings in mammalian vision. Indeed, the receptive field profiles in the retina, LGN and V1 are close to ideal to what is motivated by the idealized requirements. By complementing receptive field measurements with selection mechanisms over the parameters in the receptive field families, it is shown how *true invariance* of receptive field responses can be obtained under scaling transformations, affine transformations and Galilean transformations. Thereby, the framework provides a mathematically well-founded and biologically plausible model for how basic invariance properties can be achieved already at the level of receptive fields and support invariant recognition of objects and events under variations in viewpoint, retinal size, object motion and illumination. The theory can *explain* the different shapes of receptive field profiles found in biological vision, which are tuned to different sizes and orientations in the image domain as well as to different image velocities in space-time, from a requirement that the visual system should be invariant to the natural types of image transformations that occur in its environment.

## Introduction

We maintain a stable perception of our environment although the brightness patterns reaching our eyes undergo substantial changes. This shows that our visual system possesses invariance properties with respect to several types of image transformations:

If you approach an object, it will change its size on the retina. Nevertheless, the perception remains the same, which reflects a *scale invariance*. It is well-known that humans and other animals have functionally important invariance properties with respect to variations in scale. For example, Biederman and Cooper [1] demonstrated that reaction times for recognition of line drawings were independent of whether the primed object was presented at the same or a different size as when originally viewed. Logothetis *et al.* [2] found that there are cells in the inferior temporal cortex (IT) of monkeys for which the magnitude of the cell's response is the same whether the stimulus subtended 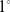 or 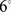 of visual angle. Ito *et al.* [3] found that about 20 percent of anterior IT cells responded to ranges of size variations greater than 4 octaves, whereas about 40 percent responded to size ranges less than 2 octaves. Furmanski and Engel [4] found that learning with application to object recognition transfers across changes in image size. The neural mechanisms underlying object recognition are rapid and lead to scale-invariant properties as soon as 100–300 ms after stimulus onset (Hung *et al.* [5]).

In a similar manner, if you rotate an object in front of you, the projected brightness pattern will be deformed on the retina, typically by different amounts in different directions. To first order of approximation, such image deformations can be modelled by local *affine transformations*, which include the effects of in-plane *rotations* and perspective *foreshortening*. For example, Logothetis *et al.* [2] and Booth and Rolls [6] have shown that in the monkey IT cortex there are both neurons that respond selectively to particular views of familiar objects as well as populations of single neurons that have view-invariant representations over different views of familiar objects. Edelmann and Bülthoff [7] have on the other hand shown that the time for recognizing unfamiliar objects from novel views increases with the 3-D rotation angle between the presented and previously seen views. Still, subjects are able to recognize unfamiliar objects from novel views, provided that the 3-D rotation is moderate.

If an object moves in front of you, it may in addition to a *translation* also lead to a time-dependent motion field in the brightness pattern on the retina. You may or you may not fixate on the object. Depending on the relative motion between the object and the observer, this motion field can to first order of approximation be modelled by local *Galilean transformations*. Regarding biological counterparts of such relative motions, Rodman and Albright [8] and Lagae *et al.* [9] have shown that in area MT of monkeys there are neurons with high selectivity to the speed and direction of visual motion over large ranges of image velocities. Petersen *et al.* [10] have shown that there are neurons in area MT that adapt their response properties to the direction and velocity of motion. Smeets and Brenner [11] have shown that reaction times for motion perception can be different for absolute and relative motion and that reaction times may specifically depend on the relative motion between the object and the background. When Einstein derived his relativity theory, he used as a basic assumption the requirement that the equations should be invariant under Galilean transformations [12].

The measured luminosity of surface patterns in the world may in turn vary over several orders of magnitude. Nevertheless we are able to preserve the identity of an object as we move it out of or into a shade, which reflects important invariance properties under *intensity transformations*. The Weber-Fechner law states that the ratio of an increment threshold 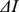 in image luminosity for a just noticeable difference in relation to the background intensity 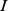 is constant over large ranges of luminosity variations (Palmer [13, pages 671–672]). The pupil of the eye and the sensitivity of the photoreceptors are continuously adapting to ambient illumination (Hurley [14]).

To be able to function robustly in a complex natural world, the visual system must be able to deal with these image transformations in an efficient and appropriate manner to maintain a stable perception as the brightness pattern changes on the retina. One specific approach is by computing *invariant features* whose values or representations remain unchanged or only moderately affected under basic image transformations. A weaker but nevertheless highly useful approach is by computing visual representations that possess suitable *covariance properties*, which means that the representations are transformed in a well-behaved and well-understood manner under corresponding image transformations. A covariant image representation can then in turn constitute the basis for computing truly invariant image representations, and thus enable invariant visual recognition processes at the systems level, in analogy with corresponding invariance principles as postulated for biological vision systems by different authors (Rolls [15]; DiCarlo and Maunsell [16]; Grimes and Rao [17]; [18]; DiCarlo and Cox [19]; Goris and Beek [20]).

The subject of this paper is to introduce a computational framework for modelling receptive fields at the earliest stages in the visual system corresponding to the retina, LGN and V1 and to show how this framework allows for *basic invariance or covariance properties of visual operations* with respect to all the above mentioned phenomena. This framework can be derived from *symmetry properties* of the natural environment (Lindeberg [21], [22]) and leads to predictions of *receptive field profiles* in good agreement with receptive measurements reported in the literature (Hubel and Wiesel [23]–[25]; DeAngelis *et al.* [26], [27]). Specifically, explicit phenomenological models will be given of LGN neurons and simple cells in V1 and be compared to related models in terms of Gabor functions (Marčelja [28]; Jones and Palmer [29], [30]), differences of Gaussians (Rodieck [31]) or Gaussian derivatives (Koenderink and van Doorn [32]; Young [33]; Young *et al.* [34], [35]). Notably, the evolution properties of the receptive field profiles in this model can be described by diffusion equations and are therefore suitable for implementation on a biological architecture, since the computations can be expressed in terms of communications between neighbouring computational units, where either a single computational unit or a group of computational units may be interpreted as corresponding to a neuron or a group of neurons. Specifically, computational models involving diffusion equations arise in mean field theory for approximating the computations that are performed by populations of neurons (Omurtag *et al.* [36]; Mattia and Guidic [37]; Faugeras *et al.* [38]).

The symmetry properties underlying the formulation of this theory will be described in the section “Model for early visual pathway in an idealized vision system” and reflect the desirable properties of an idealized vision system that (i) objects at different positions, scales and orientations in image space should be processed in a structurally similar manner, and (ii) objects should be perceived in a similar way under variations in viewing distance, viewing direction, relative motion in relation to the observer and illumination variations.

Combined with complementary *selection mechanisms* over receptive fields at different scales (Lindeberg [39]), receptive fields adapted to different affine image deformations (Lindeberg and Gårding [40]) and different Galilean motions (Lindeberg *et al.* [21], [41]), it will also be shown how true invariance of receptive field responses can be obtained with respect to local scaling transformations, affine transformations and Galilean transformations. These selection mechanisms are based on either (i) the computation of local extrema over the parameters of the receptive fields or alternatively based on (ii) comparisons of local receptive field responses to affine invariant or Galilean fixed-point requirements (to be described later). On a neural architecture, these geometric invariance properties are therefore compatible with a routing mechanism (Olshausen *et al.* [42]) that operates on the output from families of receptive fields that are tuned to different scales, spatial orientations and image velocities. In this respect, the resulting approach will bear similarity to the approach by Riesenhuber and Poggio [43], where receptive field responses at different scales are routed forward by a soft winner-take-all mechanism, with the theoretical additions that the invariance properties over scale can here be formally proven and the presented framework specifically states how the receptive fields should be normalized over scale. Furthermore, our approach extends to true and provable invariance properties under more general affine and Galilean transformations.

A direct consequence of these invariance properties established for receptive field responses is that they can be *propagated to invariance properties of visual operations at higher levels*, and thus enable invariant recognition of visual objects and events under variations in viewing direction, retinal size, object motion and illumination. In this way, the presented framework provides a computational theory for how basic invariance properties of a visual system can achieved already at the level of receptive fields. Another consequence is that the presented framework could be used for *explaining* the families of receptive field profiles tuned to different orientations and image velocities in space and space-time that have been observed in biological vision from a requirement of that the corresponding receptive field responses should be invariant or covariant under corresponding image transformations. A main purpose of this article is to provide a *synthesis* where such structural components are combined into a coherent framework for achieving basic invariance properties of a visual system and relating these results, which have been derived mathematically, to corresponding functional properties of neurons in a biological vision system.

Another major aim of this article is to try to bridge the gap between computer vision and biological vision, by demonstrating how concepts originally developed for purposes in computer vision can be related to corresponding notions in computational neuroscience and biological vision. In particular, we will argue for explicit incorporation of basic image transformations into computational neuroscience models of vision. If such image transformations are not appropriately modelled and if the model is then exposed to test data that contain image variations outside the domain of variabilities that are spanned by the training data, then an artificial neuron model may have severe problems with robustness. If on the other hand the covariance properties corresponding to the natural variabilities in the world underlying the formation of natural image statistics are explicitly modelled and if corresponding invariance properties are built into the computational neuroscience model and also used in the learning stage, we argue that it should be possible to increase the robustness of a neuro-inspired artificial vision system to natural image variations. Specifically, we will present explicit computational mechanism for obtaining true scale invariance, affine invariance, Galilean invariance and illumination invariance for image measurements in terms of local receptive field responses.

Interestingly, the proposed framework for receptive fields can be derived *by necessity* from a mathematical analysis based on symmetry requirements with respect to the above mentioned image transformations in combination with a few additional requirements concerning the internal structure and computations in the first stages of a vision system that will be described in more detail below. In these respects, the framework can be regarded as both (i) a canonical mathematical model for the first stages of processing in an idealized vision system and as (ii) a plausible computational model for biological vision. Specifically, compared to previous approaches of learning receptive field properties and visual models from the statistics of natural image data (Field [44]; van der Schaaf and van Hateren [45]: Olshausen and Field [46]; Rao and Ballard [47]; Simoncelli and Olshausen [48]; Geisler [49]) the proposed theoretical model makes it possible to determine spatial and spatio-temporal receptive fields from first principles that reflect symmetry properties of the environment and thus without need for any explicit training stage or gathering of representative image data. In relation to such learning based models, the proposed normative approach can be seen as describing the solutions that an ideal learning based system may converge to, if exposed to a sufficiently large and representative set of natural image data. The framework for achieving true invariance properties of receptive field responses is also theoretically strong in the sense that the invariance properties can be formally proven given the idealized model of receptive fields.

In their survey of our knowledge of the early visual system, Carandini *et al.* [50] emphasize the need for functional models to establish a link between neural biology and perception. More recently, Einhäuser and König [51] argue for the need for normative approaches in vision. This paper can be seen as developing the consequences of such ways of reasoning by showing how basic invariance properties of visual processes at the systems level can be obtained already at the level of receptive fields, using a normative approach.

## Model for early visual pathway in an idealized vision system

In the following we will state a number of basic requirements concerning the earliest levels of processing in an idealized vision system, which will be used for deriving *idealized functional models of receptive fields*. Let us stress that the aim is not to model specific properties of human vision or any other species. Instead the goal is to describe basic characteristics of the image formation process and the computations that are performed after the registration of image luminosity on the retina. These assumptions will then be used for narrowing down the class of possible image operations that are compatible with structural requirements, which reflect symmetry properties of the environment. Thereafter, it will be shown how this approach applies to modelling of biological receptive fields and how the resulting receptive fields can be regarded as biologically plausible.

For simplicity, we will assume that the image measurements are performed on a planar retina under perspective projection. With appropriate modifications, a corresponding treatment can be performed with a spherical camera geometry.

Let us therefore assume that the vision system receives image data that are either defined on a (i) *purely spatial domain* 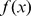 or a (ii) *spatio-temporal domain* 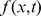 with 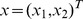. Let us regard the purpose of the earliest levels of visual representations as computing a family of *internal representations* 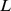 from 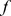, whose output can be used as input to different types of visual modules. In biological terms, this would correspond to a similar type of *sharing* as V1 produces output for several downstream areas such as V2, V4 and V5/MT.

An important requirement on these early levels of processing is that we would like them to be *uncommitted* operations without being too specifically adapted to a particular task that would limit the applicability for other visual tasks. We would also desire a *uniform structure* on the first stages of visual computations.

### Spatial (time-independent) image data

Concerning terminology, we will use the convention that a receptive field refers to a region 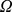 in visual space over which some computations are being performed. These computations will be represented by an operator 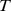, whose support region is 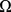. Generally, the notion of a receptive field will be used to refer to both the operator 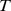 and its support region 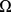. In some cases when referring specifically to the support region 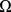 only, we will refer to it as the support region of the receptive field.

Given a purely spatial image 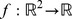, let us consider the problem of defining a family of internal representations
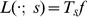
(1)


for some family of operators 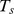 that are indexed by some parameter 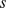, where 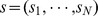 may be a multi-dimensional parameter with 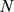 dimensions. (The dot “

” at the position of the first argument 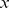 of 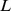 means that 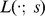 when given a fixed value of the parameter 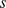 only should be regarded as a function over 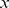.) In the following we shall state a number of structural requirements on a visual front-end as motivated by the types of computations that are to be performed at the earliest levels of processing in combination with symmetry properties of the surrounding world.

#### Linearity

Initially, it is natural to require 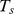 to be a *linear* operator, such that
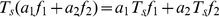
(2)holds for all functions 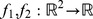 and all scalar constants 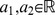. An underlying motivation to this linearity requirement is that the earliest levels of visual processing should make as few irreversible decisions as possible.

Linearity also implies that a number of special properties of receptive fields (to be described below) will transfer to spatial and spatio-temporal derivatives of these and do therefore imply that different types of image structures will be treated in a similar manner irrespective of what types of linear filters they are captured by.

#### Translational invariance

Let us also require 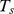 to be a *shift-invariant operator* in the sense that it commutes with the shift operator 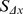 defined by 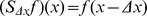, such that
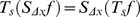
(3)holds for all 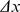. The motivation behind this assumption is the basic requirement that the perception of a visual object should be the same irrespective of its position in the image plane. Alternatively stated, the operator 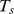 can be said to be *homogeneous across space*.

For us humans and other higher mammals, the retina is obviously not translationally invariant. Instead, finer scale receptive fields are concentrated to the fovea in such a way that the minimum receptive field size increases essentially linearily with eccentricity. With respect to such a sensor space, the assumption about translational invariance should be taken as an idealized model for the region in space where there are receptive fields above a certain size.

#### Convolution structure

Together, the assumptions of linearity and shift-invariance imply that the internal representations 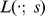 are given by *convolution transformations*

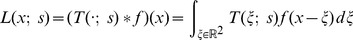
(4)where 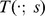 denotes some family of convolution kernels. Later, we will refer to these convolution kernels as receptive fields.

#### The issue of scale

A fundamental property of the convolution operation is that it may reflect different types of image structures depending on the spatial extent (the width) of the convolution kernel.

- Convolution with a *large support* kernel will have the ability to respond to phenomena at *coarse scales*.
- A kernel with *small support* may on the other hand only capture phenomena at *fine scales*.

From this viewpoint it is natural to associate an interpretation of *scale* with the parameter 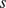 and we will assume that the limit case of the internal representations when 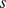 tend to zero should correspond to the original image pattern 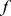


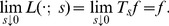
(5)


#### Semi-group structure

From the interpretation of 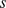 as a scale parameter, it is natural to require the image operators 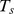 to form a *semi-group* over 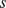


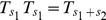
(6)with a corresponding semi-group structure for the convolution kernels



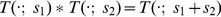
(7)such that the composition of two different receptive fields coupled in cascade will also be a member of the same receptive field family. Then, the transformation between any different and ordered scale levels 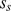 and 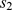 with 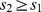 will obey the *cascade property*



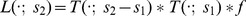


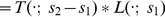
(8)
*i.e.* a similar type of transformation as from the original data 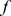. An image representation with these properties is referred to as a *multi-scale representation*.

Concerning the parameterization of this semi-group, we will in the specific case of a one-dimensional (scalar) scale parameter assume the parameter 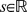 to have a direct interpretation of scale, whereas in the case of a multi-dimensional parameter 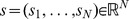, these parameters could also encode for other properties of the convolution kernels in terms of the orientation 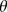 in image space or the degree of elongation 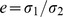, where 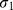 and 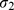 denote the spatial extents in different directions. The convolution kernels will, however, not be be required to form a semi-group over any type of parameterization, such as the parameters 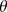 or 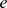. Instead, we will assume that there exists some parameterization 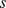 for which an additive linear semi-group structure can be defined and from which the latter types of parameters can then be derived.

#### Self-similarity over scale

Regarding the family of convolution kernels used for computing a multi-scale representation, it is also natural to require them to *self-similar over scale*, such that if 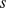 is a one-dimensional scale parameter then all the kernels correspond to rescaled copies
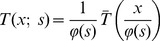
(9)


of some prototype kernel 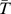 for some transformation 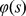 of the scale parameter. If 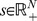 is a multi-dimensional scale parameter, the requirement of self-similarity over scale can be generalized into



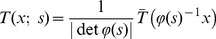
(10)where 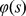 now denotes a non-singular 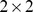-dimensional matrix regarding a 2-D image domain and 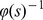 its inverse. With this definition, a multi-scale representation with a scalar scale parameter 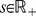 will be based on uniform rescalings of the prototype kernel, whereas a multi-scale representation based on a multi-dimensional scale parameter might also allow for rotations as well as non-uniform affine deformations of the prototype kernel.

The reason for introducing a function 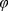 for transforming the scale parameter 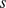 into a scaling factor 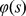 in image space, is that the requirement of a semi-group structure (6) does not imply any restriction on how the parameter 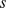 should be related to image measurements in dimensions of length — the semi-group structure only implies an abstract ordering relation between coarser and finer scales 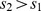 that could also be satisfied for any monotonously increasing transformation of the parameter 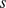. For the Gaussian scale-space concept with a scalar scale parameter and given by (24) this transformation is given by 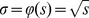, whereas for the affine Gaussian scale-space concept given by (29) it is given by the matrix square root function 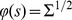, where 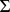 denotes the covariance matrix that describes the spatial extent and the orientation of the affine Gaussian kernels.

#### Infinitesimal generator

For theoretical analysis it is preferable if the scale parameter can be treated as a *continuous scale parameter* and if image representations between adjacent levels of scale can be related by partial differential equations. Such relations can be expressed if the semi-group possesses an *infinitesimal generator* (Hille and Phillips [52])
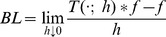
(11)and implies that image representations between adjacent levels of scale can be related by *differential evolution equations*; for a scalar scale parameter of the form



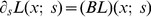
(12)for some operator 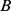 and for an 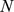-dimensional scale parameter of the form



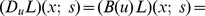


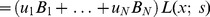
(13)for any positive direction 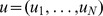 in the parameter space with 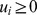 for every 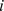. In (Lindeberg [21]) it is shown how such differential relationships can be ensured given a proper selection of functional spaces and sufficient regularity requirements over space 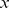 and scale 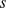 in terms of Sobolev norms. We shall therefore henceforth regard the internal representations 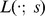 as differentiable with respect to both the image space and scale parameter(s).

#### Non-enhancement of local extrema

For the internal representations 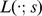 that are computed from the original image data 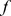 it is in addition essential that the operators 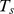 *do not generate new structures* in the representations at coarser scales that do not correspond to simplifications of corresponding image structures in the original image data.

**Figure 1:**
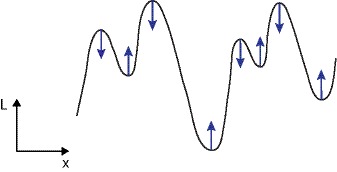
The requirement of non-enhancement of local extrema is a way of restricting the class of possible image operations by formalizing the notion that new image structures must not be created with increasing scale, by requiring that the value at a local maximum must not increase and that the value at a local minimum must not decrease.

A particularly useful way of formalizing this requirement is that *local extrema must not be enhanced with increasing scale*. In other worlds, if a point 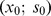 is a local (spatial) maximum of the mapping 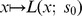 then the value must not increase with scale. Similarly, if a point 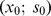 is a local (spatial) minimum of the mapping 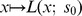, then the value must not decrease with scale. Given the above mentioned differentiability property with respect to scale, we say that the multi-scale representation constitutes a *scale-space representation* if it for a scalar scale parameter satisfies the following conditions at any non-degenerate local extremum point:
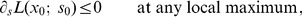
(14)

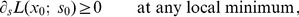
(15)or for a multi-parameter scale-space



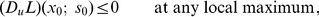
(16)

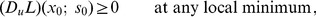
(17)for any positive direction 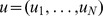 in the parameter space with 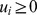 for every 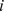 (see figure 1).

#### Rotational invariance

If we restrict ourselves to a scale-space representation based on a scalar (one-dimensional) scale parameter 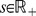, then it is natural to require the scale-space kernels to be *rotationally symmetric*

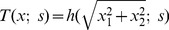
(18)


for some one-dimensional function 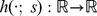. Such a symmetry requirement can be motivated by the requirement that in the absence of further information, all spatial directions should be equally treated (isotropy).

For a scale-space representation based on a multi-dimensional scale parameter, one may also consider a weaker requirement of rotational invariance at the level of a family of kernels, for example regarding a set of elongated kernels with different orientations in image space. Then, the family of kernels may capture image data of different orientation in a rotationally invariant manner, for example if all image orientations are explicitly represented or if the receptive fields corresponding to different orientations in image space can be related by linear combinations.

#### Affine covariance

The perspective mapping from surfaces of objects in the 3-D world to the 2-D image space gives rises to image deformations in the image domain. If we approximate the non-linear perspective mapping from a surface pattern in the world to the image plane by a local linear transformation (the derivative), then we can model this deformation by an *affine transformation*

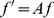
(19)corresponding to



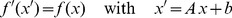
(20)where 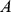 represents an affine transformation operator operating on functions and 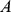 is the affine transformation matrix. To ensure that the internal representations behave nicely under image deformations, it is natural to require a possibility of relating them under affine transformations



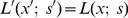
(21)corresponding to



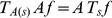
(22)for some transformation 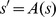 of the scale parameter. Unfortunately, it turns out that affine covariance cannot be achieved with a scalar scale parameter and linear operations. As will be shown below, it can, however, be achieved with a 3-parameter linear scale-space.

### Necessity result concerning spatial receptive fields

Given the above mentioned requirements it can be shown that if we assume (i) linearity, (ii) shift-invariance over space, (iii) semi-group property over scale, (iv) sufficient regularity properties over space and scale and (v) non-enhancement of local extrema, then the scale-space representation over a 2-D spatial domain must satisfy (Lindeberg [21, theorem 5, page 42])
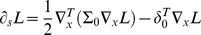
(23)for some 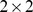 covariance matrix 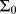 and some 2-D vector 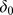 with 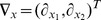. If we in addition require the convolution kernels to be *mirror symmetric* through the origin 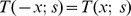 then the offset vector 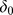 must be zero. There are two special cases within this class of operations that are particularly worth emphasizing.

#### Gaussian receptive fields

**Figure 2:**
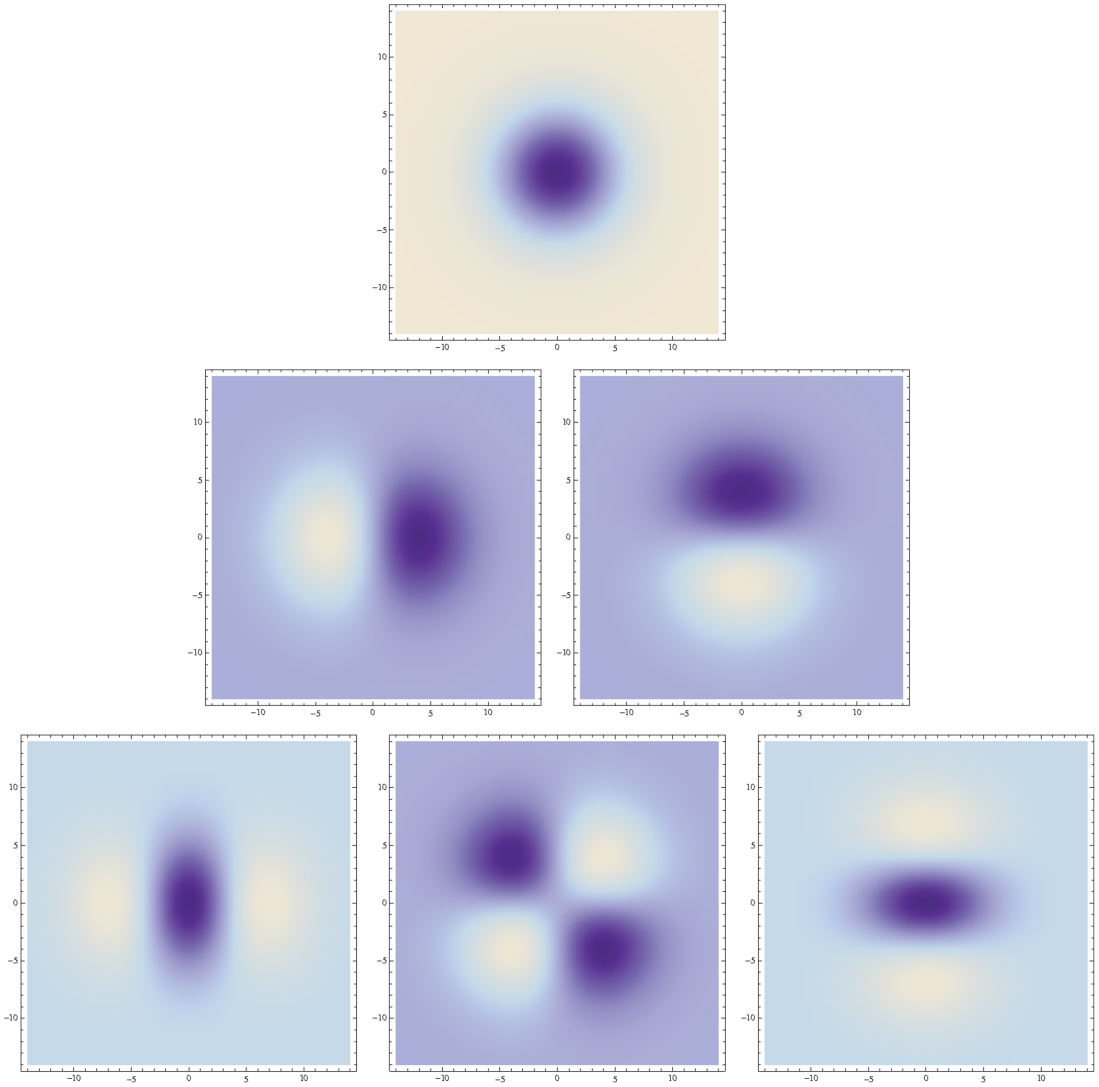
Spatial receptive fields formed by the 2-D Gaussian kernel with its partial derivatives up to order two. The corresponding family of receptive fields is closed under translations, rotations and scaling transformations.



If we require the corresponding convolution kernels to be rotationally symmetric, then it follows that they will be Gaussians
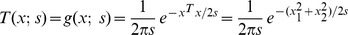
(24)with corresponding Gaussian derivative operators



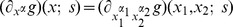


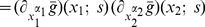
(25)(with 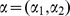 where 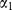 and 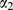 denote the order of differentiation in the 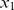
- and 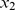-directions, respectively) as shown in figure 2 with the corresponding one-dimensional Gaussian kernel and its Gaussian derivatives of the form

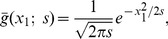
(26)

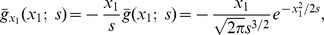


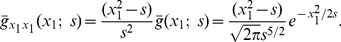
(28)


Such Gaussian functions have been previously used for modelling biological vision by Koenderink and van Doorn [32], [53]–[55] who proposed the Gaussian derivative model for visual operations and pioneered the modelling of visual operations using differential geometry, by Young [33] who showed that there are receptive fields in the striate cortex that can be well modelled by Gaussian derivatives up to order four, by Lindeberg [56] who extended the Gaussian derivative model for receptive fields with corresponding idealized discretizations and by Petitot [57], [58] who expressed a differential geometric model for illusory contours and the singularities in the orientation fields in the primary visual cortex known as pinwheels; see also Sarti *et al.* [59] for extensions of the latter model to rotations and rescalings of non-Gaussian receptive field profiles.

More generally, these Gaussian derivative operators can be used as a *general basis* for expressing image operations such as feature detection, feature classification, surface shape, image matching and image-based recognition (Witkin [60]; Koenderink [61]; Koenderink and van Doorn [62]; Lindeberg [63]–[65]; Florack [66]; ter Haar Romeny [67]); see specifically Schiele and Crowley [68], Linde and Lindeberg [69], [70], Lowe [71], and Bay *et al.* [72] for explicit approaches for object recognition based on Gaussian receptive fields or approximations thereof.

#### Affine-adapted Gaussian receptive fields

**Figure 3:**
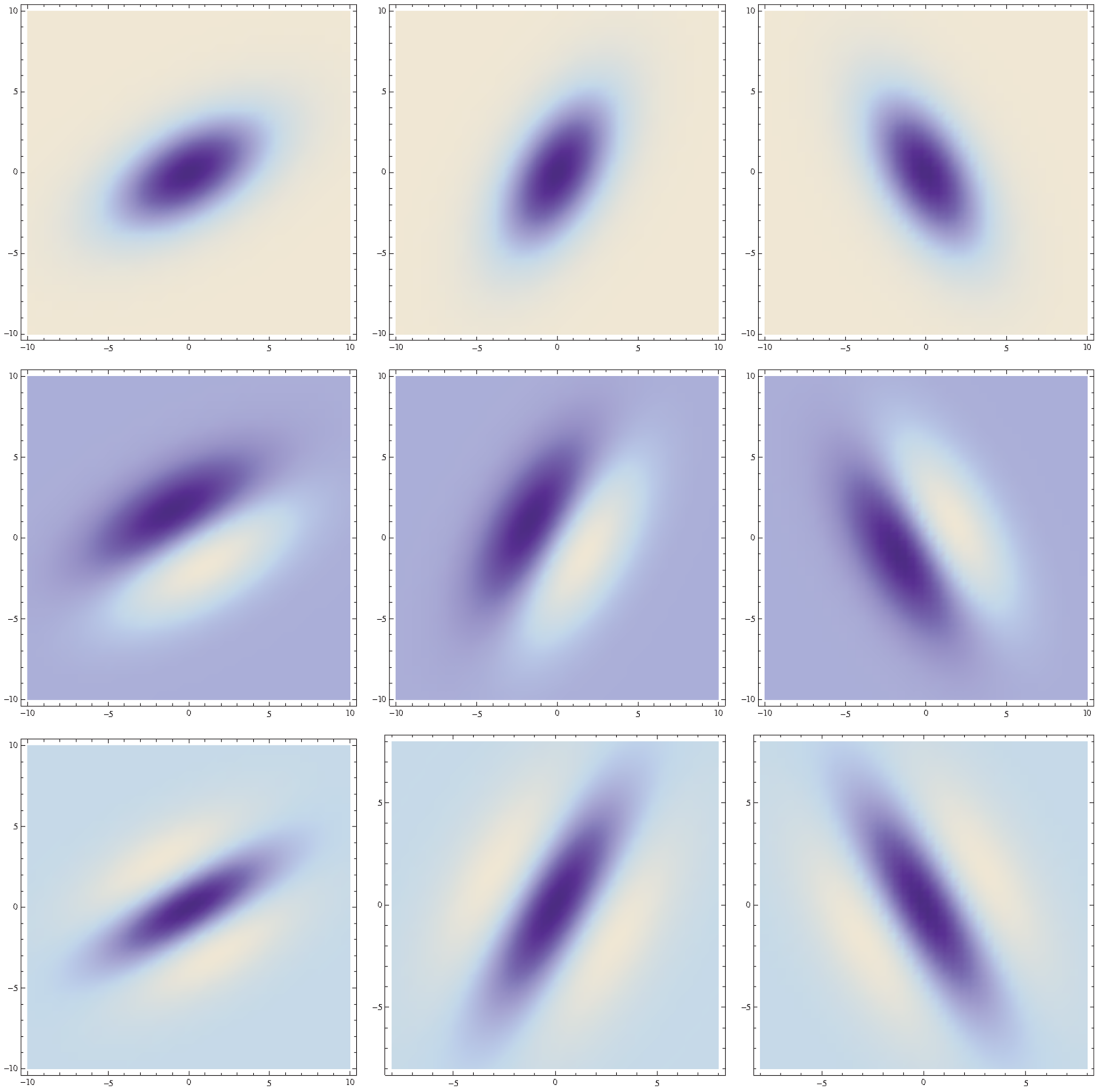
Spatial receptive fields formed by affine Gaussian kernels and directional derivatives of these. The corresponding family of receptive fields is closed under general affine transformations of the spatial domain, including translations, rotations, scaling transformations and perspective foreshortening.



If we relax the requirement of rotational symmetry and relax it into the requirement of mirror symmetry through the origin, then it follows that the convolution kernels must instead be *affine Gaussian kernels* (Lindeberg [63])
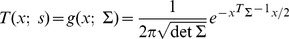
(29)where 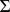 denotes any symmetric positive semi-definite 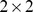 matrix. This affine scale-space concept is *closed* under affine transformations, meaning that if we for affine related images



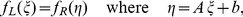
(30)(which may represent images of a local image patch seen from two different views, either by 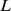 and 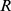 representing the left and right views of a binocular observer or 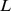 and 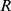 representing two different views registered by a monocular observer by translating and/or rotating the object and/or the observer) define corresponding scale-space representations 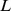 and 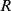 according to



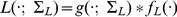
(31)




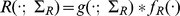
(32)


then these scale-space representations will be related according to (Lindeberg [63]; Lindeberg and Gårding [40])



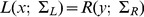
(33)where



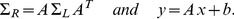
(34)In other words, given that an image 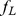 is affine transformed into an image 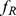 it will always be possible to find a transformation between the scale parameters 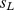 and 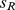 in the two domains that makes it possible to match the corresponding derived internal representations 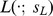 and 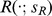. Figure 3 shows a few examples of such kernels in different directions with the covariance matrix parameterized according to
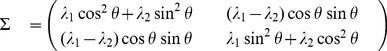
(35)with 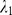 and 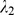 denoting the eigenvalues and 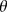 the orientation. Directional derivatives of these kernels can in turn be obtained from linear combinations of partial derivative operators according to

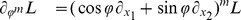


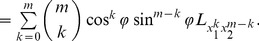
(36)


With respect to biological vision, these kernels can be used for modelling receptive fields that are oriented in the spatial domain, as will be described in connection with equation (71) in the section on “Computational modelling of biological receptive fields”. For computer vision they can be used for computing *affine invariant image descriptors* for *e.g.* cues to surface shape, image-based matching and recognition (Lindeberg [63]; Lindeberg and Gårding [40]: Baumberg [73]; Mikolajczyk and Schmid [74]; Tuytelaars and van Gool [75]; Lazebnik *et al.* [76]; Rothganger *et al.* [77]).

#### Note on receptive fields formed from derivatives of the convolution kernels

Due to the linearity of the differential equation (23), which has been derived by necessity from the structural requirements, it follows that also the result of applying a linear operator 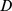 to the solution 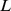 will also satisfy the differential equation, however, with a different initial condition
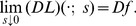
(37)


The result of applying a linear operator 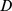 to the scale-space representation 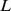 will therefore satisfy the above mentioned structural requirements of linearity, shift invariance, the weaker form of rotational invariance at the group level and non-enhancement of local extrema, with the semi-group structure (6) replaced by the cascade property
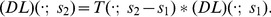
(38)


Then, one may ask if any linear operator 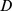 would be reasonable? From the requirement of scale invariance, however, if follows that that the operator 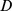 must not be allowed to have non-infinitesimal support, since a non-infinitesimal support 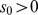 would violate the requirement of self-similarity over scale (9) and it would not be possible to perform image measurements at a scale level lower than 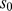. Thus, any receptive field operator derived from the scale-space representation in a manner compatible with the structural arguments must correspond to local derivatives. In the illustrations above, partial derivatives and directional derivatives up to order two have been shown.

For directional derivatives that have been derived from elongated kernels whose underlying zero-order convolution kernels are not rotationally symmetric, it should be noted that we have aligned the directions of the directional derivative operators to the orientations of the underlying kernels. A structural motivation for making such an alignment can be obtained from a requirement of a weaker form of rotational symmetry at the group level. If we would like the family of receptive fields to be rotationally symmetric as a group, then it is natural to require the directional derivative operators to be transformed in a similar way as the underlying kernels.

Receptive fields in terms of derivatives of the convolution kernels derived by necessity do also have additional advantages if one adds a further structural requirement of invariance under additive intensity transformations 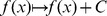. A zero-order receptive field will be affected by such an intensity transformation, whereas higher order derivatives are invariant under additive intensity transformations. As will be described in the section on “Invariance property under illumination variations”, this form of invariance has a particularly interesting physical interpretation with regard to a logarithmic intensity scale.

### Spatio-temporal image data

For spatio-temporal image data 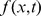 defined on a 2+1−D spatio-temporal domain with 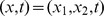 it is natural to inherit the symmetry requirements over the spatial domain. In addition, the following structural requirements can be imposed motivated by the special nature of time and space-time:

### Galilean covariance

For time-dependent spatio-temporal image data, we may have *relative motions* between objects in the world and the observer, where a constant velocity translational motion can be modelled by a *Galilean transformation*

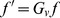
(39)corresponding to



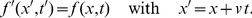
(40)To enable a consistent visual interpretation under different relative motions, it is natural to require that it should be possible to transform internal representations 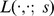 that are computed from spatio-temporal image data under different relative motions
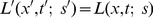
(41)corresponding to



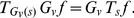
(42)Such a property is referred to as *Galilean covariance*.

#### Temporal causality

For a vision system that interacts with the environment in a real-time setting, a fundamental constraint on the convolution kernels (the spatio-temporal receptive fields) is that they cannot access data from the future. Hence, they must be *time-causal* in the sense that convolution kernel must be zero for any relative time moment that would imply access to the future:
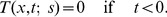
(43)


#### Time-recursivity

Another fundamental constraint on a real-time system is that it cannot keep a record of everything that has happened in the past. Hence, the computations must be based on a limited internal *temporal buffer* 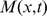, which should provide:

- a sufficient record of past information and
- sufficient information to update its internal state when new information arrives.

A particularly useful solution is to use the internal representations 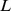 at different temporal scales also used as the memory buffer of the past. In (Lindeberg [21, section 5.1.3, page 57]) it is shown that such a requirement can be formalized by a time-recursive updating rule of the form
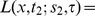


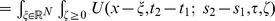


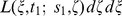


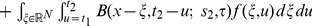
(44)which is required to hold for any pair of scale levels 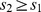 and any two time moments 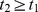, where

- the kernel 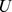 updates the internal state,
- the kernel 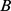 incorporates new image data into the representation,

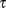
 is the temporal scale and 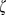 an integration variable referring to internal temporal buffers at different temporal scales.

#### Non-enhancement of local extrema in a time-recursive setting

For a time-recursive spatio-temporal visual front-end it is also natural to generalize the notion of non-enhancement of local extrema, such that it is required to hold both with respect to increasing spatial scales 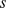 and evolution over time 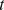. Thus, if at some spatial scale 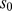 and time moment 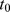 a point 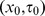 is a local maximum (minimum) for the mapping
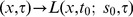
(45)then for *every positive direction* 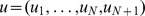 in the 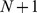-dimensional space spanned by 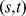, the directional derivative 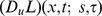 must satisfy



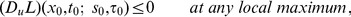
(46)

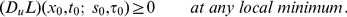
(47)


## Necessity results concerning spatio-temporal receptive fields

We shall now describe how these structural requirements restrict the class of possible spatio-temporal receptive fields.

### Non-causal spatio-temporal receptive fields

If one disregards the requirements of temporal causality and time recursivity and instead requires (i) linearity, (ii) shift invariance over space and time, (iii) semi-group property over spatial and temporal scales, (iv) sufficient regularity properties over space, time and spatio-temporal scales and (v) non-enhancement of local extrema for a multi-parameter scale-space, then it follows from (Lindeberg [21, theorem 5, page 42]) that the scale-space representation over a 2+1-D spatio-temporal domain must satisfy
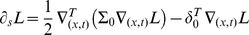
(48)for some 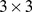 covariance matrix 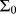 and some 3-D vector 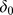 with 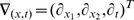.

**Figure 4:**
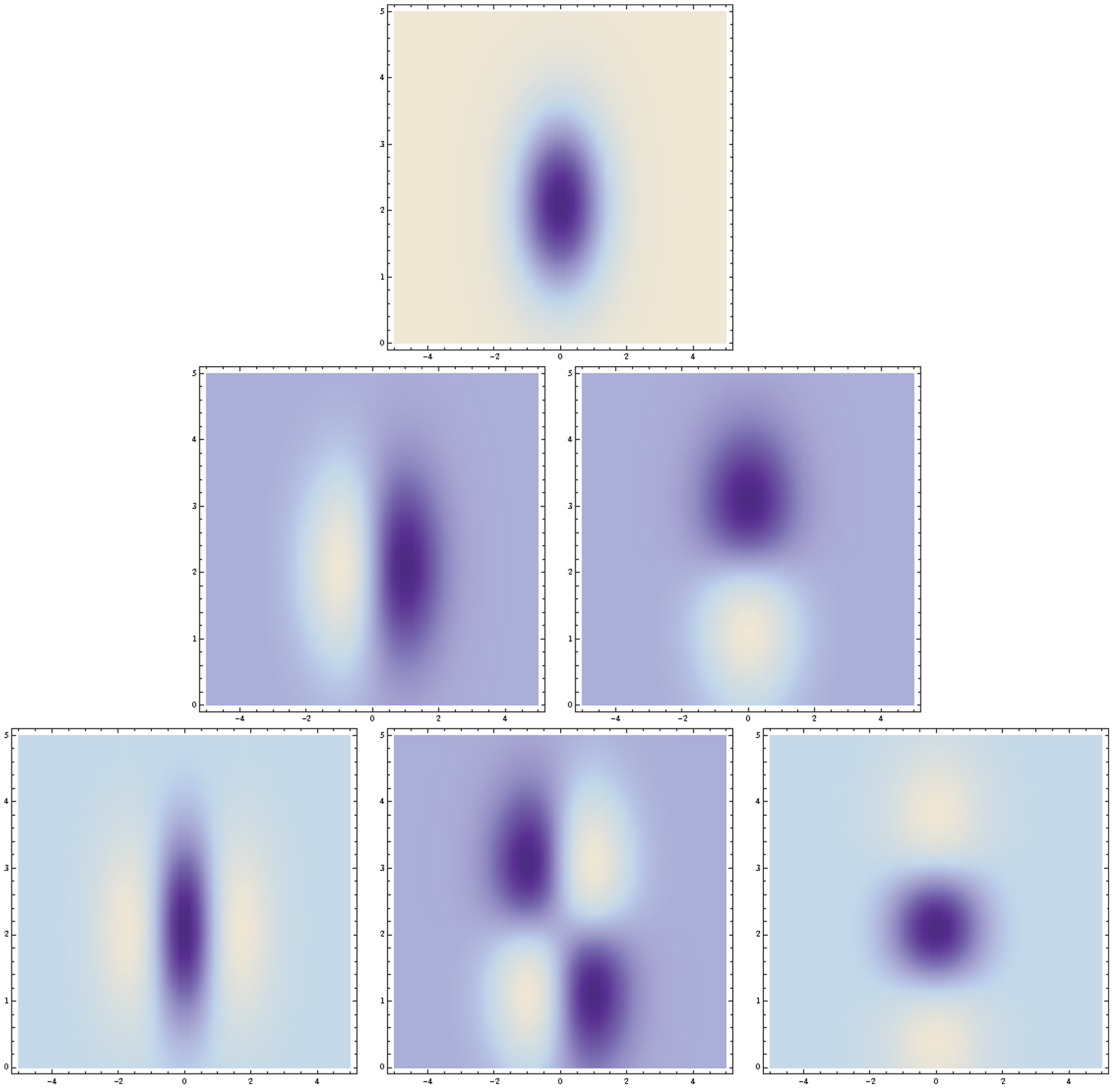
Non-causal and space-time separable spatio-temporal receptive fields over 1+1−D space-time as generated by the Gaussian spatio-temporal scale-space model with 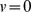. This family of receptive fields is closed under rescalings of the spatial and temporal dimensions. (Horizontal axis: space 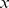. Vertical axis: time 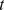.)

**Figure 5:**
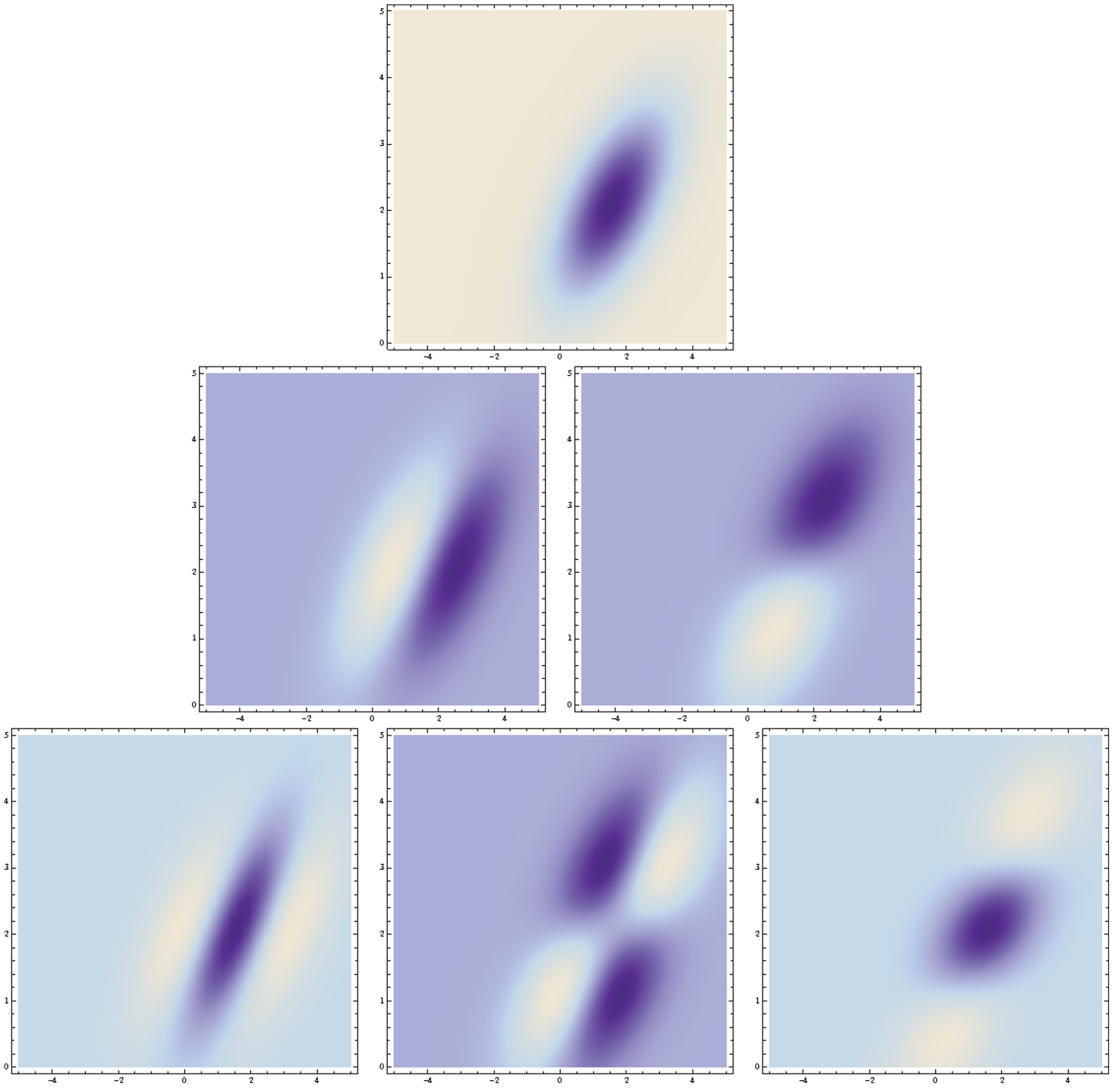
Non-causal and velocity-adapted spatio-temporal receptive fields over 1+1-D space-time as generated by the Gaussian spatio-temporal scale-space model for a non-zero image velocity 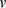
. This family of receptive fields is closed under rescalings of the spatial and temporal dimensions as well as Galilean transformations. (Horizontal axis: space 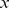. Vertical axis: time 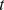.)

In terms of convolution kernels, the zero-order receptive fields will then be *spatio-temporal Gaussian kernels*

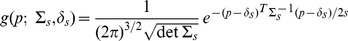
(49)with 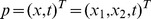,



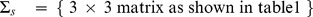
(50)




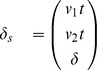
(51)


where (i) 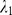, 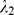 and 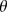 determine the *spatial extent*, (ii) 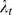 determines the *temporal extent*, (iii) 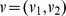 denotes the *image velocity* and (iv) 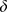 represents a *temporal delay*. From the corresponding *Gaussian spatio-temporal scale-space*



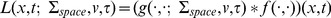
(52)spatio-temporal derivatives can then be defined according to



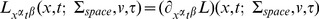
(53)with corresponding velocity-adapted temporal derivatives



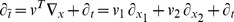
(54)as illustrated in figure 4 and figure 5 for the case of a 1+1−D space-time.

**Table 1:** Spatio-temporal covariance matrix for the Gaussian spatio-temporal scale-space used for modelling non-causal spatio-temporal receptive fields in equation (50).

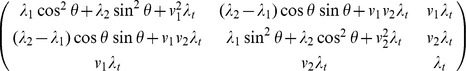

Motivated by the requirement of Galilean covariance, it is natural to align the directions 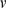 in space-time for which these velocity-adapted spatio-temporal derivatives are computed to the velocity values used in the underlying zero-order spatio-temporal kernels, since the resulting velocity-adapted spatio-temporal derivatives will then be Galilean covariant. Such receptive fields can be used for modelling spatio-temporal receptive fields in biological vision (Lindeberg [21], [56]; Young *et al.* [34], [35]) and for computing spatio-temporal image features and Galilean invariant image descriptors for spatio-temporal recognition in computer vision (Laptev and Lindeberg [78]–[80]; Laptev *et al.* [81]; Willems *et al.* [82]).

#### Transformation property under Galilean transformations

Under a Galilean transformation of space-time (40), in matrix form written
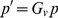
(55)corresponding to
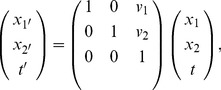
(56)the corresponding Gaussian spatio-temporal representations are related in an algebraically similar way (30–33) as the affine Gaussian scale-space with the affine transformation matrix 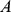 replaced by a Galilean transformation matrix 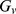. In other words, if two spatio-temporal image patterns 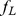 and 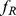 are related by a Galilean transformation encompassing a translation 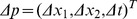 in space-time
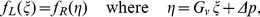
(57)(which may represent time-dependent image data registered of an object under different relative motions between the object and the observer) and if corresponding spatio-temporal scale-space representations 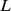 and 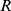 of 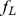 and 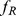 are defined according to



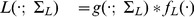
(58)

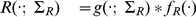
(59)for general spatio-temporal covariance matrices 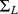 and 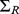 of the form (50), then these spatio-temporal scale-space representations will be related according to
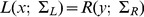
(60)where



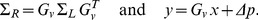
(61)Given two spatio-temporal image patterns that are related by a Galilean transformation, such as arising when an object is observed with different relative motion between the object and the viewing direction of the observer, it will therefore be possible to perfectly match the spatio-temporal receptive field responses computed from the different spatio-temporal image patterns. Such a perfect matching would, however, not be possible without velocity adaptation, *i.e.*, if the spatio-temporal receptive fields would be computed using space-time separable receptive fields only.

### Time-causal spatio-temporal receptive fields

If we on the other hand with regard to real-time biological vision want to respect both temporal causality and temporal recursivity, we obtain a different family of *time-causal* spatio-temporal receptive fields. Given the requirements of (i) linearity, (ii) shift invariance over space and time, (iii) temporal causality, (iv) time-recursivity, (v) semi-group property over spatial scales 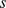 and time 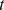 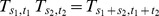, (vi) sufficient regularity properties over space, time and spatio-temporal scales and (vii) non-enhancement of local extrema in a time-recursive setting, then it follows that the time-causal spatio-temporal scale-space must satisfy the *system* of diffusion equations (Lindeberg [21, equations (88–89), page 52, theorem 17, page 78])
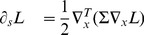
(62)

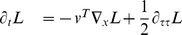
(63)for some 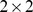 spatial covariance matrix 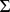 and some image velocity 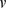 with 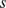 denoting the *spatial scale* and 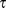 the *temporal scale*. In terms of receptive fields, this spatio-temporal scale-space can be computed by convolution kernels of the form



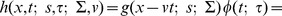


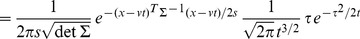
(64)where

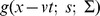
 is a *velocity-adapted 2-D affine Gaussian kernel* with covariance matrix 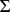 and

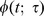
 is a *time-causal smoothing kernel over time* with temporal scale parameter 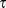.

**Figure 6:**
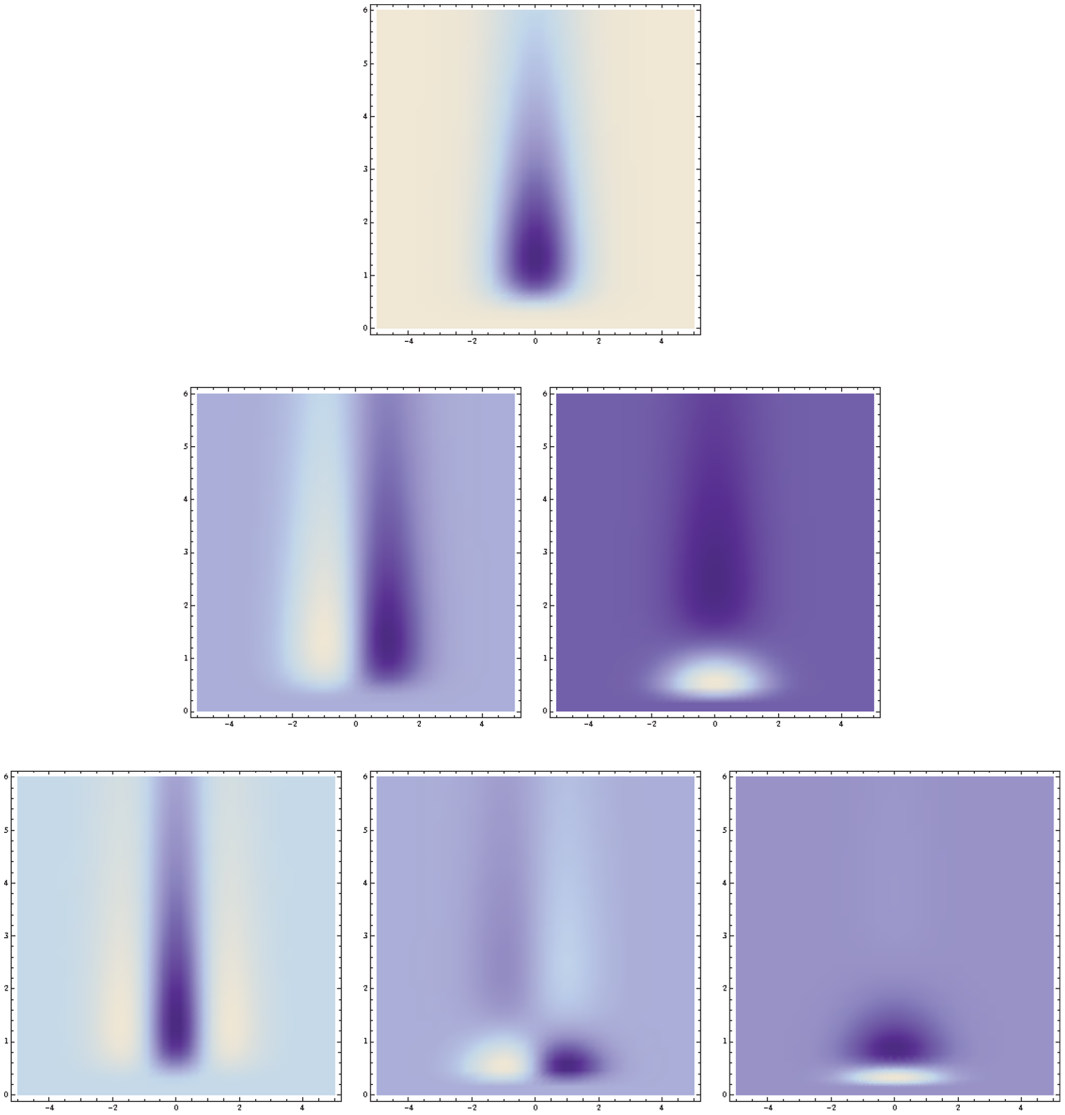
Time-causal and space-time separable spatio-temporal receptive fields over a 1+1−D space-time as generated by the time-causal spatio-temporal scale-space model with 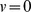. This family of receptive fields is closed under rescalings of the spatial and temporal dimensions. (Horizontal axis: space 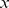. Vertical axis: time 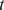.)

**Figure 7:**
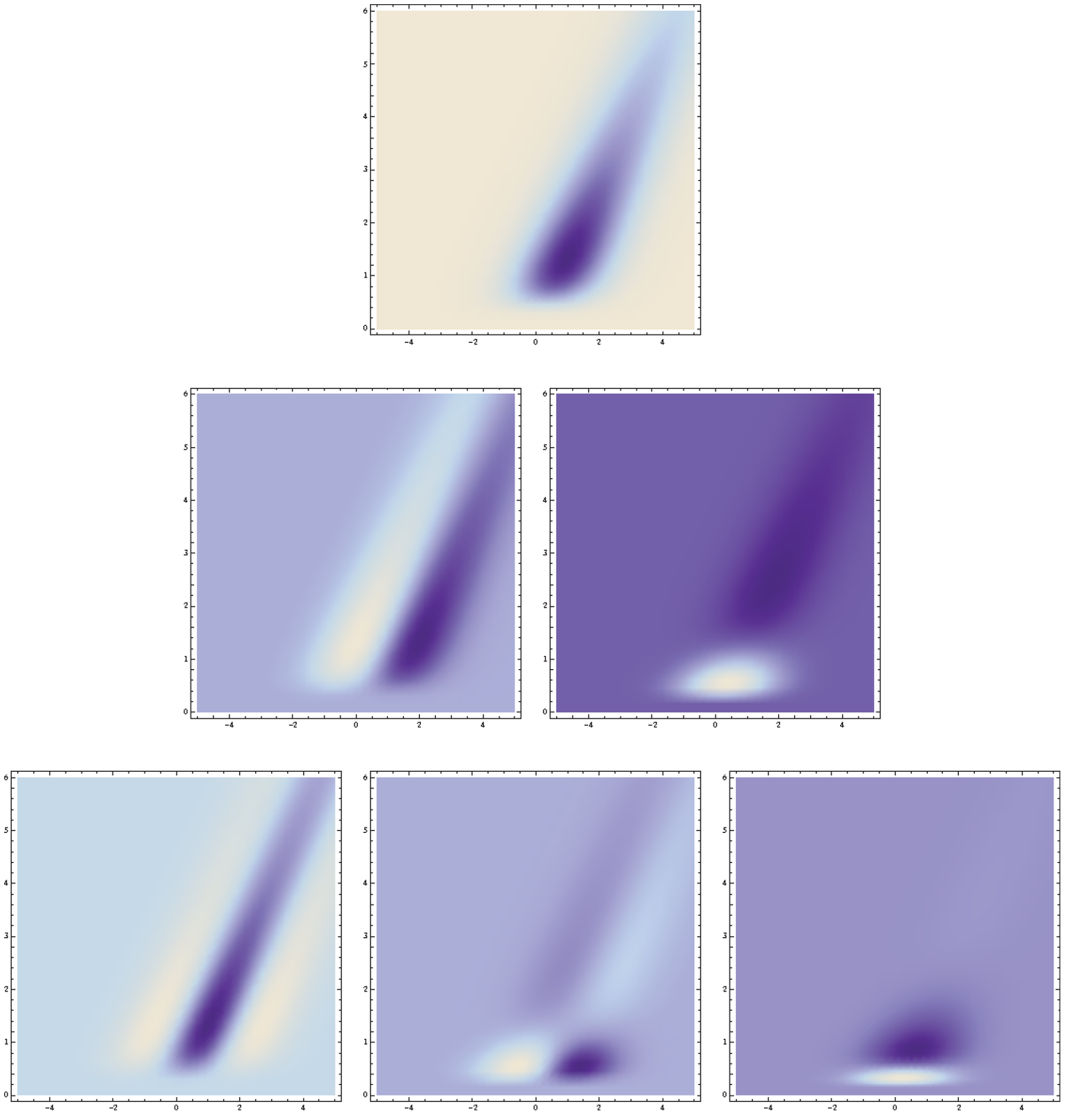
Time-causal and velocity-adapted spatio-temporal receptive fields over a 1+1−D space-time as generated by the time-causal spatio-temporal scale-space model with 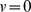
. This family of receptive fields is closed under rescalings of the spatial and temporal dimensions as well as Galilean transformations. (Horizontal axis: space 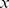. Vertical axis: time 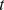.)

From these kernels, spatio-temporal partial derivatives and velocity-adapted derivatives can be computed in a corresponding manner (53) and (54) as for the Gaussian spatio-temporal scale-space concept; see figure 6 and figure 7 for illustrations in the case of a 1+1−D space-time.

Concerning the relations between the non-causal spatio-temporal model in section "Non-causal spatio-temporal receptive fields", and the time-causal model in section "Time-causal spatio-temporal receptive fields", please note that requirement of non-enhancement of local extrema is formulated in different ways in the two cases: (i) For the non-causal scale-space model, the condition about non-enhancement condition is based on points that are local extrema with respect to both space 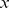 and time 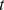. At such points, a sign condition is imposed on the derivative in any positive direction over spatial scales 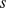 and temporal scale 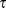. (ii) For the time-causal scale-space model, the notion of local extrema is based on points that are local extrema with respect to space 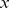 and the internal temporal buffers at different temporal scales 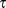. At such points, a sign condition is imposed on the derivatives in the parameter space defined by the spatial scale parameters 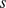 and time 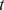. Thus, in addition to the restriction to time-causal convolution kernels (43) the derivation of the time-causal scale-space model is also based on different structural requirements.

Other time-causal temporal scale-space models have been proposed by Koenderink [83] based on a logarithmic transformation of time in relation to a time delay relative to the present moment and by Lindeberg and Fagerström [84] based on a set of first-order integrators corresponding to truncated exponential filters with time constants 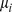 coupled in cascade
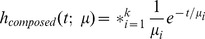
(65)with the composed kernel having temporal variance



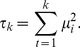
(66)Such first-order temporal integrators satisfy weaker scale-space properties in the sense of guaranteeing non-creation of local extrema or zero-crossings for a one-dimensional temporal signal, although they do not permit true covariance under rescalings of the temporal axis. Moreover, they are inherently time recursive and obey a temporal update rule between adjacent temporal scale levels 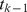 and 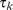 of the following form:
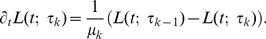
(67)


Such kernels can also be used as an idealized computational model for temporal processing in biological neurons (Koch [85, Chapters 11–12]). If we combine these purely temporal smoothing kernels with the general form of spatio-temporal kernels
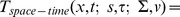


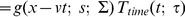
(68)as obtained from a principled treatment over the joint space-time domain, we obtain an additional class of time-causal and time-recursive spatio-temporal receptive fields with the additional restrictions that the temporal scale parameter has to be discretized already in the theory and that temporal covariance cannot hold exactly for temporal scale levels that have been determined beforehand. For the logarithmic scale-time approach by Koenderink [83], there is, however, not any known time-recursive implementation suitable for real-time processing.

## Computational modelling of biological receptive fields

An attractive property of the presented framework for early receptive fields is that it generates receptive field profiles in good agreement with receptive field profiles found by cell recordings in the retina, LGN and V1 of higher mammals. DeAngelis *et al.* [26] and DeAngelis and Anzai [27] present overviews of (classical) receptive fields in the *joint* space-time domain. As outlined in (Lindeberg [21, section 6]), the Gaussian and time-causal scale-space concepts presented here can be used for generating predictions of receptive field profiles that are qualitatively very similar to *all* the spatial and spatio-temporal receptive fields presented in these surveys.

### LGN neurons

In the LGN, most cells (i) have approximately *circular-center surround* and most receptive fields are (ii) *space-time separable* (DeAngelis *et al.* [26]; DeAngelis and Anzai [27]). A corresponding idealized scale-space model for such receptive fields can be expressed as
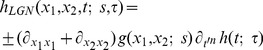
(69)where

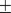
 determines the polarity (on-center/off-surround *vs*: off-center/on-surround),

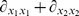
 denotes the spatial Laplacian operator,

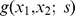
 denotes a rotationally symmetric spatial Gaussian,

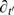
 denotes a temporal derivative operator with respect to a possibly self-similar transformation of time 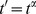 or 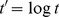 such that 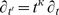 for some constant 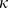 [21, section 5.1, pages 59–61],

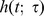
 is a temporal smoothing kernel over time corresponding to the time-causal smoothing kernel 
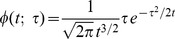
 in (64) or a non-causal time-shifted Gaussian kernel 
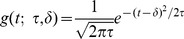
 according to (49), alternatively a time-causal kernel of the form (65) corresponding to a set of first-order integrators over time coupled in cascade,

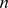
 is the order of temporal differentiation,

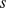
 is the spatial scale parameter and

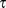
 is the temporal scale parameter.

**Figure 8:**
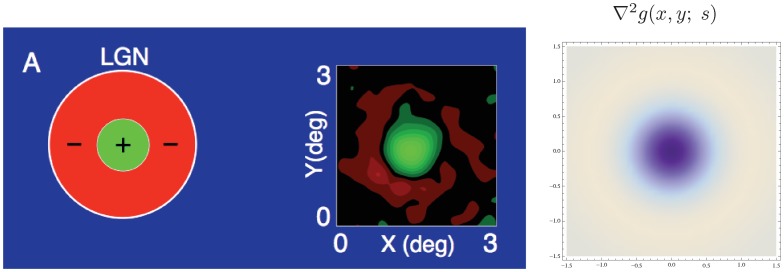
Spatial component of receptive fields in the LGN. (left) Receptive fields in the LGN have approximately circular center-surround responses in the spatial domain, as reported by DeAngelis *et al.* [26]. (right) In terms of Gaussian derivatives, this spatial response profile can be modelled by the Laplacian of the Gaussian 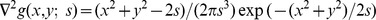, here with 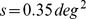.

Figure 8 shows a comparison between the spatial component of a receptive field in the LGN with a Laplacian of the Gaussian. This model can also be used for modelling on-center/off-surround and off-center/on-surround receptive fields in the retina.

Regarding the spatial domain, the model in terms of spatial Laplacians of Gaussians 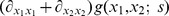 is closely related to differences of Gaussians, which have previously been shown to be good approximation of the spatial variation of receptive fields in the retina and the LGN (Rodieck [31]). This property follows from the fact that the rotationally symmetric Gaussian satisfies the isotropic diffusion equation
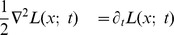


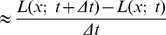


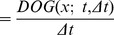
(70)which implies that differences of Gaussians can be interpreted as approximations of derivatives over scale and hence to Laplacian responses.

### Simple cells in V1

In V1 the receptive fields are generally different from the receptive fields in the LGN in the sense that they are (i) *oriented in the spatial domain* and (ii) *sensitive to specific stimulus velocities* (DeAngelis *et al.* [26]; DeAngelis and Anzai [27]).

### Spatial dependencies

**Figure 9:**
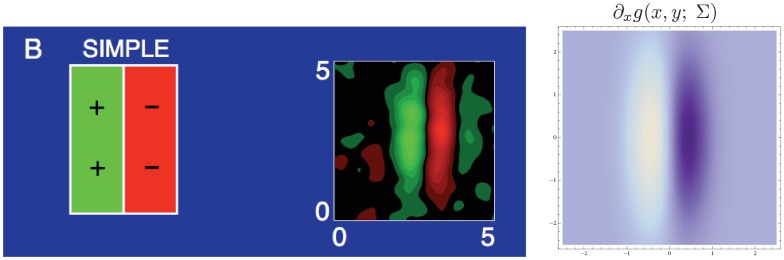
Spatial component of receptive fields in V1. (left) Simple cells in the striate cortex do usually have strong directional preference in the spatial domain, as reported by DeAngelis *et al.* [26]. (right) In terms of Gaussian derivatives, first-order directional derivatives of anisotropic affine Gaussian kernels, here aligned to the coordinate directions 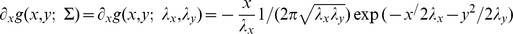 and here with 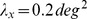 and 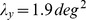, can be used as a model for simple cells with a strong directional preference.

We can express a scale-space model for the *spatial component* of this orientation dependency according to
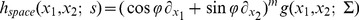
(71)where

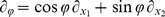
 is a directional derivative operator,

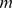
 is the order of spatial differentiation and

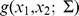
 is an affine Gaussian kernel with spatial covariance matrix 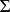 as can be parameterized according to (35)

where the direction 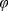 of the directional derivative operator should preferably be aligned to the orientation 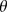 of one of the eigenvectors of 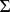. Figure 9 shows a comparison between this idealized receptive field model over the spatial domain and the spatial response properties of a simple cell in V1.

In the specific case when the covariance matrix is proportional to a unit matrix 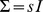, with 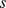 denoting the spatial scale parameter, these directional derivatives correspond to regular Gaussian derivatives as proposed as a model for spatial receptive fields by Koenderink and van Doorn [32], [62]. The use of non-isotropic covariance matrices does on the other hand allow for a higher degree of orientation selectivity. Moreover, by having a family of affine adapted kernels tuned to a family of covariance matrices with different orientations and different ratios between the scale parameters in the two directions, the family as a whole can represent affine covariance which makes it possible to perfectly match corresponding receptive field responses between different views obtained under variations of the viewing direction in relation to the object.

**Figure 10:**
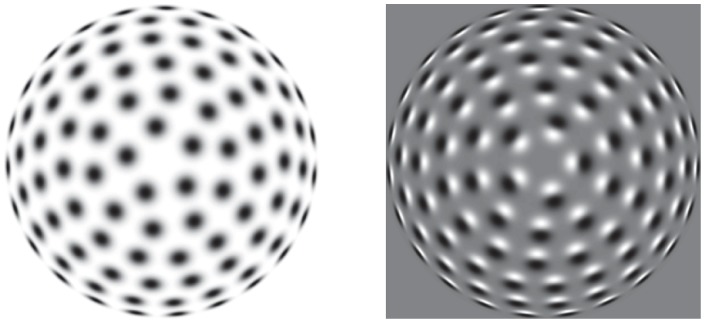
Affine Gaussian receptive fields generated for a set of covariance matrices 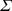 that correspond to an approximately uniform distribution on a hemisphere in the 3-D environment, which is then projected onto a 2-D image plane. (left) Zero-order receptive fields. (right) First-order receptive fields.

Figure 10 shows illustrations of affine receptive fields of different orientations and degrees of elongation as they arise if we assume that the set of all 3-D objects in the world have an approximately uniform distribution of surface orientations in 3-D space and if we furthermore assume that we observe these objects from a uniform distribution of viewing directions that are not directly coupled to properties of the objects.

This idealized model of elongated receptive fields can also be extended to recurrent intracortical feedback mechanisms as formulated by Somers *et al.* [86] and Sompolinsky and Shapley [87] by starting from the equivalent formulation in terms of the non-isotropic diffusion equation (23)
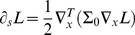
(72)with the covariance matrix 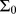 locally adapted to the statistics of image data in a neighbourhood of each image point; see Weickert [88] and Almansa and Lindeberg [89] for applications of this idea to the enhancement of local directional image structures in computer vision.

By the use of locally adapted feedback, the resulting evolution equation does not obey the original linearity and shift-invariance (homogeneity) requirements used for deriving the idealized affine Gaussian receptive field model, if the covariance matrices 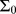 are determined from a properties of the image data that are determined in a non-linear way. For a fixed set of covariance matrices 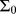 at any image point, the evolution equation will still be linear and will specifically obey non-enhancement of local extrema. In this respect, the resulting model could be regarded as a simplest form of non-linear extension of the idealized receptive field model.

#### Relations to modelling by Gabor functions

Gabor functions have been frequently used for modelling spatial receptive fields (Marčelja [28]; Jones and Palmer [29], [30]), motivated by their property of minimizing the uncertainty relation. This motivation can, however, be questioned on both theoretical and empirical grounds. Stork and Wilson [90] argue that (i) only complex-valued Gabor functions that cannot describe single receptive field minimize the uncertainty relation, (ii) the real functions that minimize this relation are Gaussian derivatives rather than Gabor functions and (iii) comparisons among Gabor and alternative fits to both psychophysical and physiological data have shown that in many cases other functions (including Gaussian derivatives) provide better fits than Gabor functions do.

Conceptually, the ripples of the Gabor functions, which are given by complex sine waves, are related to the ripples of Gaussian derivatives, which are given by Hermite functions. A Gabor function, however, requires the specification of a scale parameter and a frequency, whereas a Gaussian derivative requires a scale parameter and the order of differentiation. With the Gaussian derivative model, receptive fields of different orders can be mutually related by derivative operations, and be computed from each other by nearest-neighbour operations. The zero-order receptive fields as well as the derivative based receptive fields can be modelled by diffusion equations, and can therefore be implemented by computations between neighbouring computational units.

In relation to invariance properties, the family of affine Gaussian kernels is closed under affine image deformations, whereas the family of Gabor functions obtained by multiplying rotationally symmetric Gaussians with sine and cosine waves is not closed under affine image deformations. This means that it is not possibly to compute truly affine invariant image representations from such Gabor functions. Instead, given a pair of images that are related by a non-uniform image deformation, the lack of affine covariance implies that there will be a systematic bias in image representations derived from such Gabor functions, corresponding to the difference between the backprojected Gabor functions in the two image domains. If using receptive profiles defined from directional derivatives of affine Gaussian kernels, it will on the other hand be possible to compute affine invariant image representations.

In this respect, the Gaussian derivative model can be regarded as simpler, it can be related to image measurements by differential geometry, be derived axiomatically from symmetry principles, be computed from a minimal set of connections and allows for provable invariance properties under non-uniform (affine) image deformations. Young [33] has more generally shown that spatial receptive fields in cats and monkeys can be well modelled by Gaussian derivatives up to order four.

### Spatio-temporal dependencies

To model spatio-temporal receptive fields in the *joint space-time domain*, we can then state scale-space models of simple cells in V1 using either

- *non-causal Gaussian spatio-temporal derivative kernels*

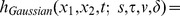


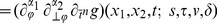
(73)

- *time-causal spatio-temporal derivative kernels*

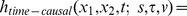


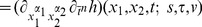
(74)with the non-causal Gaussian spatio-temporal kernels 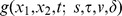 according to (49), the time-causal spatio-temporal kernels 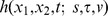 according to (64) and spatio-temporal derivatives 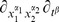 or velocity-adapted derivatives 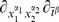 of these according to (53) and (54).

For a general orientation of receptive fields with respect to the spatial coordinate systems, the receptive fields in these scale-space models can be jointly described in the form
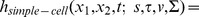


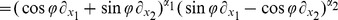


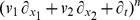


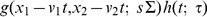
(75)where

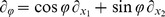
 and 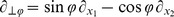 denote spatial directional derivative operators according to (36) in two orthogonal directions 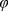 and 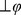,

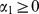
 and 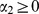 denote the orders of differentiation in the two orthogonal directions in the spatial domain with the overall spatial order of differentiation 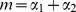,

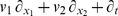
 denotes a velocity-adapted temporal derivative operator,

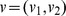
 denotes the image velocity,

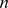
 denotes the order of temporal differentiation,

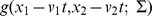
 denotes a spatial affine Gaussian kernel according to (29) that translates with image velocity 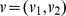 in space-time,

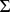
 denotes a spatial covariance matrix that can be parameterized by two eigenvalues 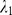 and 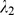 as well as an orientation 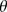 of the form (35),

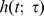
 is a temporal smoothing kernel over time corresponding to the time-causal smoothing kernel 
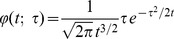
 in (64) or a non-causal time-shifted Gaussian kernel 
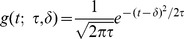
 according to (49), alternatively a time-causal kernel of the form (65) corresponding to a set of first-order integrators over time coupled in cascade,

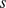
 denotes the spatial scale and

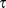
 denotes the temporal scale.

**Figure 11:**
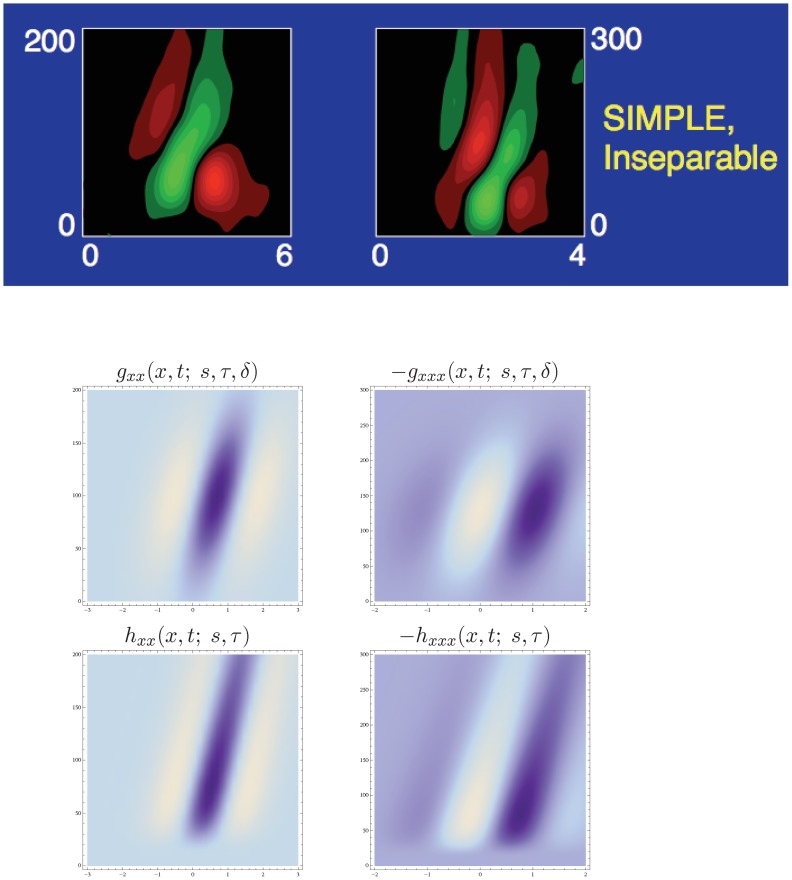
Non-separable spatio-temporal receptive fields in V1. (top row) Examples of non-separable spatio-temporal receptive field profiles in the striate cortex as reported by DeAngelis *et al.* [26]: (top left) a receptive field reminiscent of a second-order derivative in tilted space-time (compare with the left column in figure 11) (top right) a receptive reminiscent of a third-order derivative in tilted space-time (compare with the right column in figure 11). (middle and bottom rows) Non-separable spatio-temporal receptive fields obtained by applying velocity-adapted second- and third-order derivative operations in space-time to spatio-temporal smoothing kernels generated by the spatio-temporal scale-space concept. (middle left) Gaussian spatio-temporal kernel 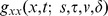 with 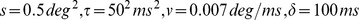. (middle right) Gaussian spatio-temporal kernel 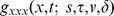 with 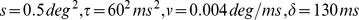. (lower left) Time-causal spatio-temporal kernel 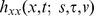 with 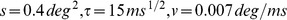. (lower right) Time-causal spatio-temporal kernel 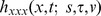 with 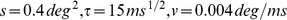. (Horizontal dimension: space 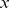. Vertical dimension: time 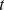.)

Figure 11 shows examples of non-separable spatio-temporal receptive fields measured by cell recordings in V1 with corresponding velocity-adapted spatio-temporal receptive fields obtained using the Gaussian scale-space and the time-causal scale-space; see also Young *et al.* [34] and Young and Lesperance [35] for a closely related approach based on Gaussian spatio-temporal derivatives although using a different type of parameterization and Lindeberg [56] for closely related earlier work. These scale-space models should be regarded as *idealized functional and phenomenological models of receptive fields* that predict how computations occur in a visual system and whose actual realization can then be implemented in different ways depending on available hardware or wetware.

Work has also been performed on learning receptive field properties and visual models from the statistics of natural image data (Field [44]; van der Schaaf and van Hateren [45]; Olshausen and Field [46]; Rao and Ballard [47]; Simoncelli and Olshausen [48]; Geisler [49]) and been shown to lead to the formation of similar receptive fields as found in biological vision. The proposed theoretical model on the other hand makes it possible to determine such receptive fields from theoretical first principles that reflect symmetry properties of the environment and thus without need for any explicit training stage or selection of representative image data. This normative approach can therefore be seen as describing the solution that an idealized learning based system may converge to, if exposed to a sufficiently large and representative set of natural image data.

An interesting observation that can be made from the similarities between the receptive field families derived by necessity from the assumptions and receptive profiles found by cell recordings in biological vision, is that receptive fields in the retina, LGN and V1 of higher mammals are very close to *ideal* in view of the stated structural requirements/symmetry properties (Lindeberg [22]). In this sense, biological vision can be seen as having adapted very well to the transformation properties of the outside world and the transformations that occur when a three-dimensional world is projected to a two-dimensional image domain.

## Mechanisms for obtaining true geometric invariances

An important property of the above mentioned families of spatial and spatio-temporal receptive fields is that they obey basic *covariance properties* under

- *rescalings* of the spatial and temporal dimensions,
- *affine transformations* of the spatial domain and
- Galilean transformations of space-time;

see (Lindeberg [21, section 5.1.2, page 56]) for more precise statements and explicit equations. These properties do in turn allow the vision system to handle:

- image data acquired with different spatial and temporal *sampling rates*, including image data that are sampled with different *spatial resolution* on a foveated sensor with decreasing sampling rate towards the periphery and spatio-temporal events that occur at *different speed* (fast *vs.* slow),
- image structures of different spatial and/or temporal *extent*, including objects of different *size* in the world and events with longer or shorter *duration* over time,
- objects at different *distances* from the camera,
- the linear component of *perspective deformations* (*e.g.* perspective foreshortening) corresponding to objects or events viewed from different viewing directions and
- the linear component of *relative motions* between objects or events in the world and the observer.

In these respects, the presented receptive field models ensure that visual representations will be *well-behaved* under *basic geometric transformations* in the image formation process.

This framework can then in turn be used as a basis for defining *truly invariant representations*. In the following, we shall describe basic approaches for this that have been developed in the area of computer vision, and have been demonstrated to be powerful mechanisms for achieving scale invariance, affine invariance and Galilean invariance for real-world data. Since these mechanisms are expressed at a functional level of receptive fields, we propose that corresponding mechanisms can be applied to neural models and a for providing a mathematically well-founded framework for explaining invariance properties in computational models.

### Scale invariance

Given a set of receptive fields that operate over some range of scale, a general approach for obtaining scale invariance is by performing *scale selection* from local extrema over scale of *scale-normalized derivatives* (Lindeberg [39], [63])
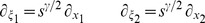
(76)where 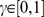 is a free parameter that can be adjusted to the task and in some cases be chosen as 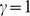. Specifically, it can be shown that if a spatial image 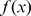 has a local extremum over scale at scale 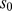 for some position 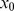 in image space, then if we define a rescaled image 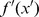 by 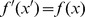 where 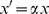 for some scaling factor 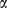, then there will be a corresponding local extremum over scale in the rescaled image 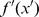 at scale 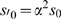 and position 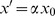 (Lindeberg [63, section 13.2.1] [39, section 4.1]). In other words, local extrema over scale of scale-normalized derivatives are preserved under scaling transformations and follow the scale variations in an appropriate manner. This property also extends to linear and non-linear combinations of receptive field responses that correspond to spatial and spatio-temporal derivatives of the Gaussian spatial and spatio-temporal scale-space concepts described in the section “Model for early visual pathway in an idealized vision system” as well as to the idealized models of biological receptive fields presented in the section “Computational modelling of biological receptive fields”.

**Figure 12:**
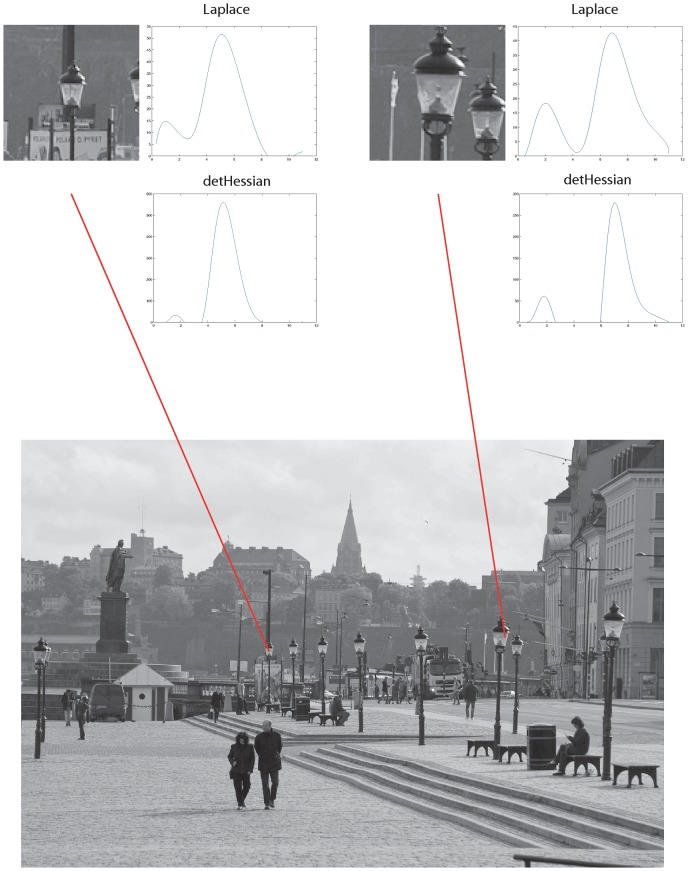
Illustration of how *scale selection* can be performed from receptive field responses by computing scale-normalized Gaussian derivative operators at different scales and then detecting local extrema over scale. Here, so-called *scale-space signatures* have been computed at the centers of two different lamps at different distances to the observer. Notice how the local extrema over scale are assumed at coarser scales for the nearby lamp than for the distant lamp. When measured in units of dimension length, the ratio between these scale estimates agrees with the ratio between the sizes of the projected lamps in the image domain.

Figure 12 illustrates this idea by performing local scale selection at two different points in a spatial image from local extrema over scale of the scale-normalized Laplacian 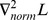 and the scale-normalized determinant of the Hessian 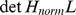 computed from Gaussian-derivative receptive fields at different spatial scales
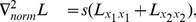
(77)

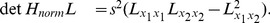
(78)


From the graphs in this figure, which show the variation over scale of the scale-normalized Laplacian 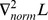 and the scale-normalized determinant of the Hessian 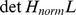 as function of effective scale 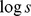, it can be seen that the local extrema over scale are assumed at a finer scale for the distant object and at a coarser scale for the nearby object. The ratio between these scale values measured in units of the standard deviation 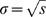 of the underlying Gaussian kernels corresponds to the ratio between the sizes of the projections between of the two lamps in the image domain and reflects the ratio between the distances between these objects and the observer.

Computing image descriptors at scale levels obtained from a scale selection step based on local extrema over scale of scale-normalized receptive field responses or equivalently computing image descriptors from local image patches that have been scale normalized with respect to such size estimates constitutes a very general approach for obtaining true *scale invariance* and has been successfully applied for different tasks in computer vision (Lindeberg [65], [91]) including scale-invariant tracking and object recognition (Bretzner and Lindeberg [92]; Lowe [71]; Bay *et al.* [72]) and estimation of time to collision from temporal size variations in the image domain (Lindeberg and Bretzner [93]; Negre *et al.* [94]).

**Figure 13:**
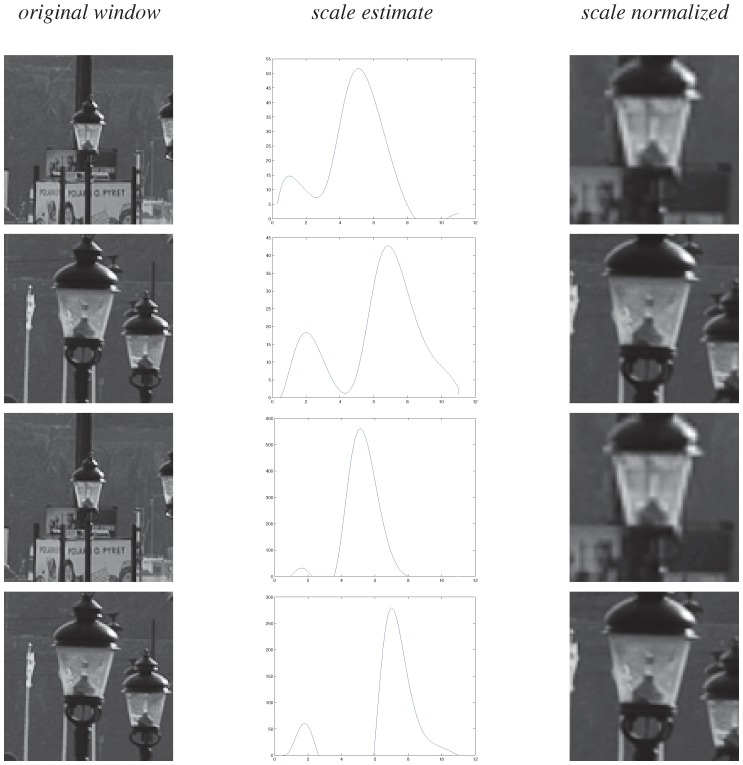
Illustration of how *scale normalization* can be performed by rescaling local image structures using scale information obtained from a scale selection mechanism. Here, the two windows selected in figure 12 have been transformed to a common *scale-invariant reference frame* by normalizing them with respect to the scale levels at which the scale-normalized Laplacian and the scale-normalized determinant of the Hessian respectively assumed their global extrema over scale. Note the similarities of the resulting scale normalized representations, although they correspond to physically different objects in the world.

Figure 13 illustrates an application of the latter scale normalization approach applied to the two windows in figure 12, by first detecting local extrema over scale of the scale-normalized Laplacian 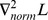 and the scale-normalized determinant of the Hessian 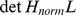 and then using these scale values for rescaling the two windows to a common reference frame. In theory any image measurement derived from the common reference frame will be truly scale invariant. Scale selection performed in this way does hence constitute a very general principle for achieving scale invariance for image measurements in terms of receptive fields.

It should be emphasized, however, that there is in principle no need for carrying out the image warping in practice as it has been done in figure 13 for the purpose of illustration. On a neural architecture it may be more efficient to consider a routing mechanism (Olshausen *et al.* [42]; Wiskott [95]) that operates on image representations at different scales and selects visual representations from the scales at which image features assume their extremum responses over scale. In this respect, the resulting model will be qualitatively rather similar to the approach by Riesenhuber and Poggio [43], where a SoftMax operation (a soft winner-take-all mechanism) is applied for computing receptive field representations at successively higher layers in a hierarchical architecture. Specifically, the notion of scale-normalized derivatives according to (76) determines how the receptive field responses as modelled by Gaussian derivatives should be normalized between different scale levels in such a model. Due to the scale covariant nature of the underlying receptive fields, it follows that the visual representations that are routed forward by the maximum selection mechanism will be truly scale invariant. Concerning the possible biological implementation of such a maximum operation, Gawne and Martin [96] have shown that there are neurons in area V4 of monkey that respond to two simultaneously presented stimuli that are well predicted by the maximum of the response to each stimulus presented separately.

(In practice, the scale normalazation in equation (77) with 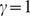 corresponds to normalizing the underlying Gaussian derivative receptive fields (25) to constant 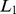-norm over scale, whereas other values of 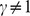 correspond to other 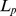-norms being constant over scale [39, section 9.1, pages 107–108].) 

### Affine invariance

Given a set of spatial receptive fields as generated from affine Gaussian kernels (29) with their directional derivatives (36) for different spatial extents and orientations as specified by different covariance matrices (35), the vision system will be faced with the task of interpreting the output from the corresponding family of receptive field responses. For example, if we assume that the vision system observes a local surface patch in the world, one may ask if some specific selection of filter parameters would be particularly suitable for interpreting the data in any given situation. Specifically, if the vision system observes the same surface patch from two different viewing directions, it would be valuable if the vision system could maintain a stable perception of the surface patch although it will be deformed in different ways in the two perspective projections onto the different image planes.

One way of selecting filter responses from such a family of affine receptive fields is by using image measurements in terms of the *second-moment matrix* (structure tensor)
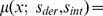


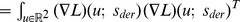


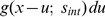
(79)where 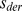 is a local scale parameter describing the scale at which spatial derivatives are computed and 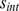 is a second integration scale parameter over which local statistics of spatial derivatives is accumulated (Lindeberg [63]; Lindeberg and Gårding [40]). These statistics correspond to weighted averages of the non-linear combinations of partial derivatives 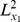, 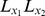 and 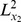 using a Gaussian function as weight, and could on a biological architecture be performed in a visual area that is based on input from V1, for example in V2.

A useful property of the second-moment matrix is that it transforms in a suitable way under affine transformations, as will be described next. If we consider an affine extension of the second-moment matrix by replacing the scalar scale parameters 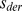 and 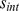 in (79) by corresponding covariance matrices 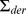 and 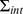


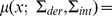


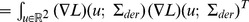


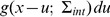
(80)and consider two images 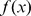 and 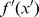 that are related by an affine transformation 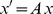 such that 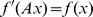, then the corresponding affine second-moment matrices will be related according to



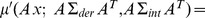


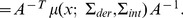
(81)


Specifically, if we can determine covariance matrices 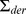 and 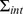 such that 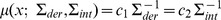 for some constants 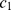 and 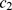, we obtain a *fixed-point* that will be *preserved under affine transformations* (Lindeberg [63]; Lindeberg and Gårding [40]). This property can be used for signalling if the image measurements that have been performed for a particular setting of filter parameters in a family of affine Gaussian receptive fields satisfy the fixed-point requirement. If so, they can be used for defining an *affine invariant reference frame* by transforming the local image patch with a linear transformation proportional to 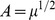.

If the local image pattern is weakly isotropic in the sense that the second-moment computed in the tangent plane of the surface is proportional to the unit matrix 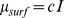 for some constant 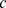 (Gårding and Lindeberg [97]), then the foreshortening caused by the perspective foreshortening will be compensated for by the affine transformation given by (81). For non-isotropic image patterns with 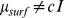 this interpretation no longer holds, but the affine transformed surface pattern will still be affine invariant.

It should be noted, however, that the affine transformation 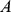 is not uniquely determined by the fixed-point requirement (80), which only determines two of the four parameters, corresponding to amount and direction of perspective foreshortening of a local surface pattern, in other words the viewing direction in relation to an object centered coordinate system. The two remaining degrees of freedom correspond to (i) an overall scaling factor corresponding the viewing distance, which can be determined by scale selection as described in the section on “Scale invariance”, and (ii) a free rotation angle, corresponding to the selection of a representative direction in the image plane. If the vertical direction is preserved under the perspective transformation, we may therefore not need to determine it or just adjust it from an initial estimate.

**Figure 14:**
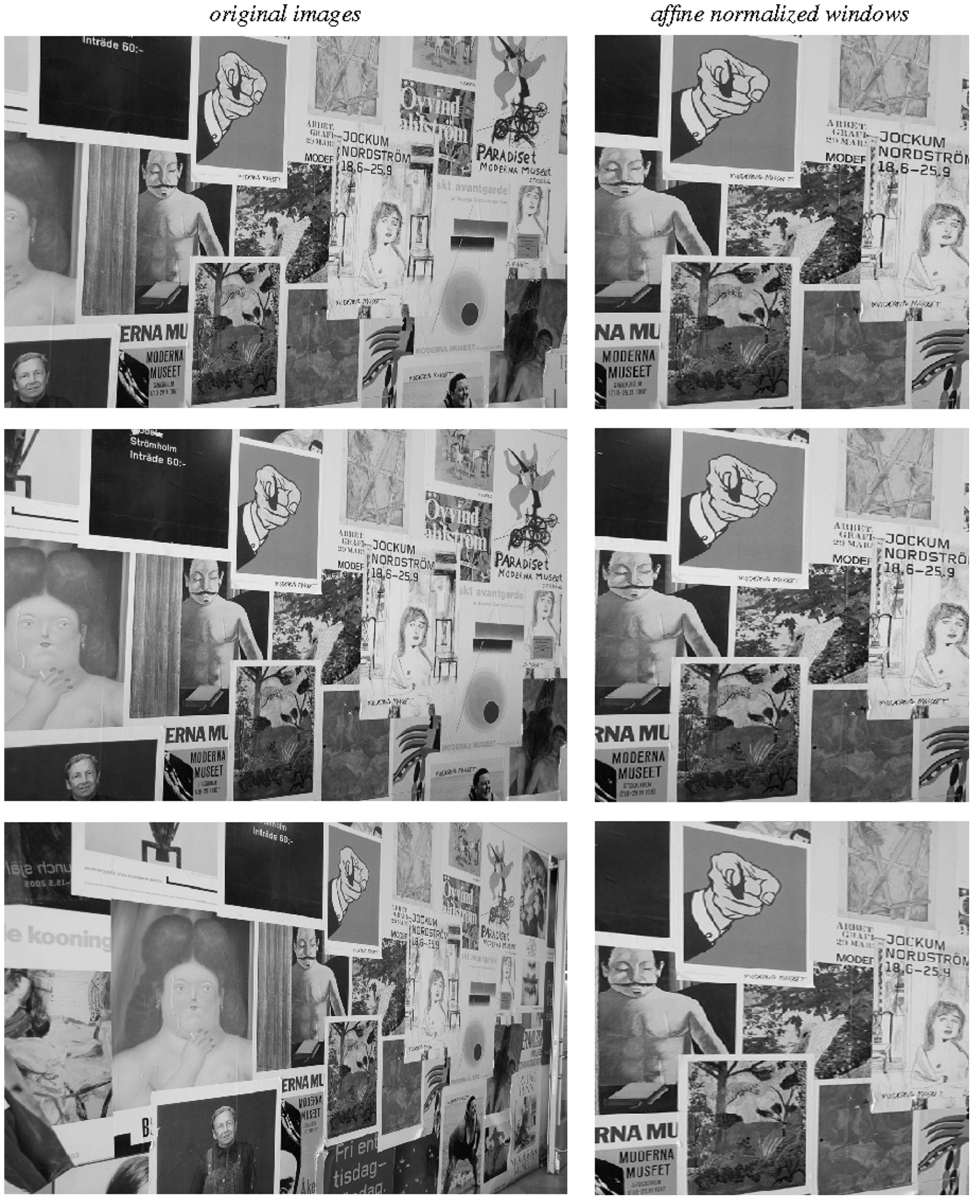
Illustration of how *affine invariance* can be achieved by normalization to an *affine invariant reference frame* determined from a second-moment matrix. The left column shows three views of a wall at Moderna Museet in Stockholm with different amount of perspective foreshortening due to variations in the viewing direction relative to the surface normal of the wall. The right column shows the result of performing affine normalization of a window in each image independently (with the windows centered at corresponding image points on the wall) using a series of affine transformations proportional to 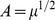 until an affine invariant fixed-point of (81) has been reached. Notice how this leads to a major compensation for the perspective foreshortening effects, which can be used for significantly improving the performance of methods for image matching and object recognition under perspective projection. With regard to receptive fields, the use of an affine family of receptive field profiles makes it possible to define image operations in the image domain that are equivalent to the use of receptive fields based on rotationally symmetric smoothing operations in an affine invariant reference frame.

Figure 14 illustrates an application of this idea to three images taken as different oblique views of a wall covered with posters. Here, a second-moment matrix 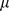 has been computed in each one of three corresponding windows in the original images shown in the left column. Then, an affine transformation matrix 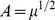 has been used for warping each window to an individual reference frame, and this so-called *affine shape-adaptation* process has been repeated until the second-moment matrix in the reference frame is sufficiently close to proportional to the unit matrix 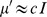. The middle figure shows the result of warping each such window in the original image to the resulting affine normalized reference frame. Due to the affine invariant property of the fixed point (81), any receptive field response computed in this reference frame will be affine invariant up to an undetermined scaling factor and a free rotation angle. Hence, this method provides a way of normalizing receptive field responses with respect to image transformations outside the similarity group that correspond to variations in the viewing direction relative to the object.

Again it should be emphasized that it is in principle not needed to perform the actual image transformation in reality to achieve the affine invariant property. On a neural architecture that computes a family of affine receptive fields with different orientations and spatial extents in parallel, one can again consider a routing mechanism that selects the receptive field responses from those receptive fields whose measurements of second-moment matrices are in best agreement with the underlying covariance matrices in relation to the fixed-point property. Then, up to a known transformation whose parameters can be computed from the corresponding second-moment matrix, these routed receptive field responses will also be affine invariant.

In the area of computer vision, this idea of affine shape adaptation has been used for defining affine invariant image descriptors with successful applications to image matching, recognition and estimation of cues to surface shape (Lindeberg and Gårding [40]; Baumberg [73]; Mikolajczyk and Schmid [74]; Tuytelaars and van Gool [75]; Lazebnik *et al.* [76]: Rothganger *et al.* [77]). The affine generalization of the SIFT operator proposed by Morel and Yu [98] is essentially also based on similar notions as underlying the affine shape adaption concept.

In the area of computer vision, other types of non-linear affine covariant evolution schemes have also been developed for curves by Sapiro and Tennenbaum [99] and Mokhtarian and Abbasi [100] and for grey-level images by Alvarez *et al.* [101]. Compared to the three-parameter affine Gaussian scale-space model for receptive fields described here, these evolution schemes achieve affine covariance by a single one-dimensional scale parameter, while sacrificing linearity. This means that the scale-space properties of the zero-order smoothed grey-level images are not guaranteed to transfer to corresponding derivatives, which on the other hand holds for receptive fields defined from the affine Gaussian scale-space. The focus of these non-linear evolution schemes is instead on preserving the properties of curves or level sets (Caselles *et al.* [102]).

On a neural architecture, one can also conceive that a neuron or a group of neurons that are adapted to a particular shape of the covariance matrix corresponding to an orientation in space could determine if the local image measurements that have been performed for this particular orientation in space are in agreement with the fixed-point requirement (81). If so, the neuron(s) could respond with a high activity if the local image measurements agree with the filter parameters to which the receptive fields are tuned and with a low activity otherwise. Hence, this framework allows for the formulation of affine invariant receptive field responses, to support view-invariant recognition at the level of groups of oriented receptive fields over a set of different covariance matrices 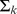.

### Galilean invariance

**Figure 15:**
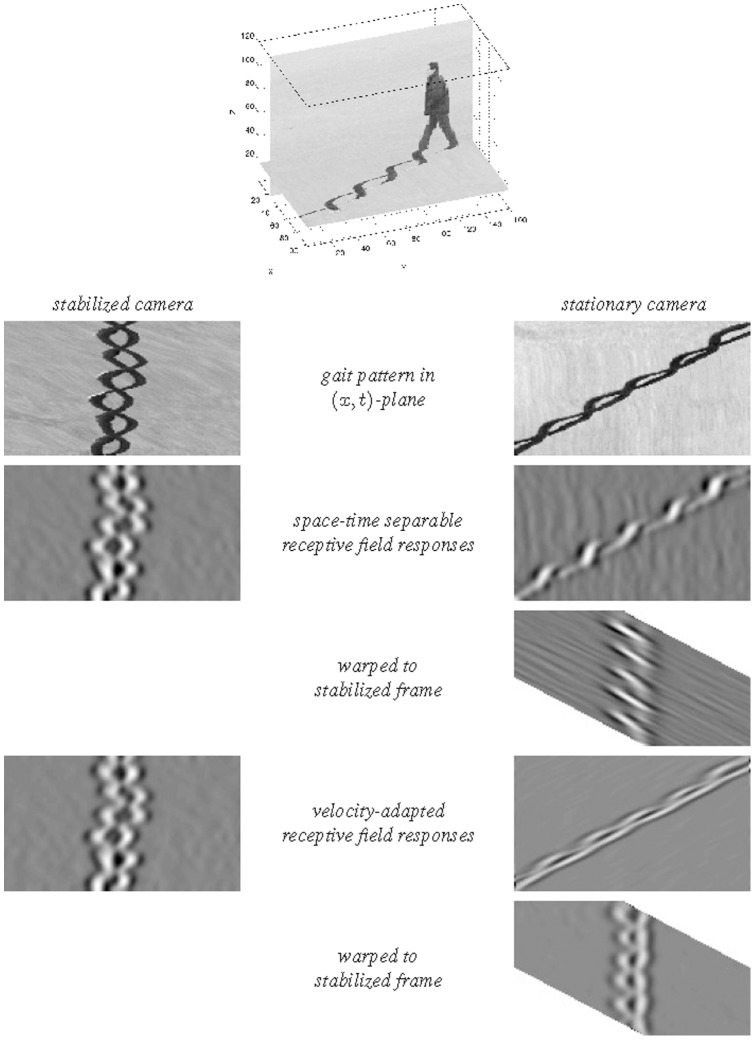
Illustration of how receptive field responses may be affected by unknown relative motions between objects in the world and the observer and of how this effect can be handled by *velocity adaptation*. The first row shows space-time traces of a walking person taken with (left column) a stabilized camera with the viewing direction following the motion of the person and (right column) a stationary camera with a fixed viewing direction for a video sequence used for the experiments in Laptev and Lindeberg [79]. The second row shows Laplacian receptive field responses computed in the two domains from space-time separable receptive fields without velocity adaptation. In the third row, these receptive field responses from the stationary camera have been space-time warped to the reference frame of the stabilized camera. As can be seen from the data, the receptive field responses are quite different in the two domains, which implies problems if one would try to match them. Hence, spatio-temporal recognition based on space-time separable receptive fields only can be a rather difficult problem. In the fourth row, the receptive field responses have instead been computed with regional velocity adaptation that aligns the space-time orientation of the receptive fields to a regional velocity estimate. In the fifth row, the velocity-adapted receptive responses from the stationary camera have been space-time warped to the reference frame of the stabilized camera. As can be seen from a comparison with the corresponding result obtained for the non-adapted receptive field responses in the third row, the use of velocity adaptation implies a better stability of receptive field responses under unknown relative motions between objects in the world and the observer. For simplicity of illustration, the velocity estimates used for velocity adaptation have here been computed *regionally* over a central region of the spatio-temporal volume containing the spatio-temporal gait pattern. In Laptev and Lindeberg [79] a corresponding *local* method for velocity adaptation is presented, where the velocity estimates for velocity adaptation are instead computed locally from extremum responses of Laplacian receptive field responses over different image velocities and spatio-temporal scales for each point in space-time.

Given a family of spatio-temporal receptive field that are adapted to motions of different image velocities 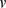 and given an object that moves with some unknown image velocity 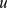 in relation to the viewing direction, the vision system also faces the problem of how to interpret the output from the family of receptive fields. Figure 15 shows an illustration of how receptive field responses may be affected by relative motions between objects in the world and the observer.

If we would know the image velocity 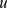 of the object beforehand, it could of course be preferable to select receptive field responses from the receptive fields that are adapted to precisely this image velocity 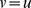. A priori, we cannot, however, assume such knowledge, since one of the basic tasks in relation to object recognition may be to determine the image velocity of an unknown moving object. There are also classes of composed spatio-temporal events consisting of different image velocities at different positions 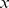 and time moments 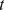 in space-time, for which it may not be trivial how a representative image velocity could be defined for the spatio-temporal event as a whole. Hence, this problem warrants a principled treatment.

Given spatio-temporal image data 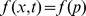 with a position in space-time denoted by 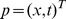, let us define a Gaussian spatio-temporal scale-space representation 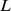 of 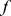 by convolution with a Gaussian spatio-temporal kernel 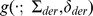 with spatio-temporal covariance matrix 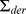 of the form (50) and with time delay 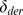. With a *spatio-temporal second-moment matrix* 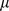 over 2+1-D space-time defined according to (Lindeberg [21, equation (191), page 73])
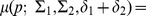


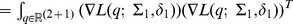


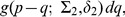
(82)where 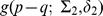 denotes a second-stage Gaussian smoothing with covariance matrix 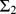 and time delay 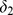 over space-time, it is indeed possible to perform such velocity selection.

Consider two Galilean-related spatio-temporal image data sets 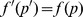 that are related by a relative image velocity 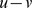 such that 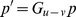 for a Galilean transformation matrix 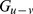 according to (40). Then, it can be shown that the corresponding spatio-temporal covariance matrices are related according to (Lindeberg [21, equation (193), page 73])
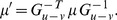
(83)


Let us introduce the notion of *Galilean diagonalization*, which corresponds to finding the unique Galilean transformation that transforms the spatio-temporal second-moment matrix to block diagonal form with all mixed purely spatio-temporal components being zero 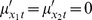 (Lindeberg *et al.* [41])
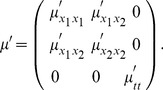
(84)


Such a block diagonalization can be obtained if the velocity vector 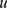 satisfies
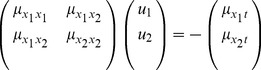
(85)with the solution
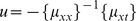
(86)i.e., structurally similar equations as are used for computing optic flow according to the method by Lukas and Kanade [103]. It can then be shown that the property of Galilean block diagonalization is preserved under Galilean transformations (Lindeberg [21, appendix C.4, pages 73–74]). Specifically, the velocity vector associated with the Galilean transformation, that brings a second-moment matrix into block diagonal form, is additive under superimposed Galilean transformations. This is a very general approach for normalizing local spatio-temporal image patterns, which also applies to spatio-temporal patterns that cannot be modelled by a Galilean transformation of an otherwise temporally stationary spatial pattern. Specifically, spatio-temporal receptive field responses that can be expressed with respect to such a spatio-temporal reference frame will be Galilean invariant.

These ideas have been applied in computer vision for performing spatio-temporal recognition under unknown relative motions between the spatio-temporal events and the observer (Laptev and Lindeberg [79], [81]). Notably the approach in [79] is based on a set of spatio-temporal receptive fields over which simultaneous selection of image velocities and spatio-temporal scales is performed.

Again, it is not necessary to carry out the spatio-temporal normalization in practice to achieve Galilean invariance. On a neural architecture based on a family of spatio-temporal receptive fields that operate over some set of image velocities in parallel, one may consider a routing mechanism that selects receptive field responses by judging the degree of agreement with the criterion of Galilean diagonalization (84) and then giving priority to the responses that are most consistent with this criterion. Notably, such a computational mechanism will have the ability to respond to different motions at different spatial and temporal scales and may therefore have the ability to handle transparent motion.

Please, note that all information that is needed for computing the spatio-temporal second-moment matrix and the Galilean diagonalization are spatio-temporal averages of the non-linear combinations 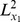, 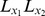, 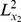, 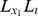, 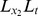 and 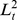 of first-order spatio-temporal derivatives and can hence be computed from spatio-temporal receptive fields. On a biological architecture, the corresponding information could therefore be computed from the output of V1 neurons in combination with an additional layer of spatio-temporal smoothing. Thus, similar type of information could in principle be computed by a visual motion area with direct access to the output from V1, such as V5/MT.

On a neural architecture, one can also conceive that a neuron or a group of neurons that are adapted to a particular image velocity could determine if the local spatio-temporal image measurements that have been performed for this particular image velocity in space-time are in agreement with the fixed-point requirement (84) of Galilean diagonalization. If so, the neuron(s) could respond with a high activity if the local measurements agree with the filter parameters to which the receptive fields are tuned and with a low activity otherwise. Hence, this framework allows for the formulation of Galilean invariant neurons, to support invariant recognition of visual objects under unknown relative motions between the object and the observer, provided that this invariance property is formulated at the level of groups of oriented receptive fields over a set of image velocities 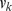.

## Invariance property under illumination variations

In the treatment so far, we have described how image measurements in terms of receptive fields are related to the geometry of space and space-time, under the assumption that the actual image intensities from which the receptive field responses are to be computed have been given beforehand. One may, however, consider alternative ways of parameterizing the intensity domain by monotonous intensity transformations that preserve the ordering between the image intensities, and in this respect would contain essentially equivalent information.

Given the huge range of luminosity variations under natural imaging conditions (corresponding to a range of the order of 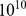 between the darkest and brightest cases for human vision), it is natural to represent the image luminosities on a *logarithmic luminosity scale*

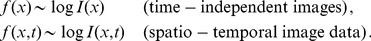
(87)


Specifically, receptive field responses that are computed from such a logarithmic parameterization of the image luminosities can be *interpreted physically* as a superposition of relative variations of surface structure and illumination variations. Given a (i) perspective camera model extended with (ii) a thin circular lens for gathering incoming light from different directions and (iii) a Lambertian illumination model extended with (iv) a spatially varying albedo factor for modelling the light that is reflects from surface patterns in the world, it can be shown (Lindeberg [22]) that a spatial receptive field response
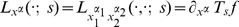
(88)of the image data 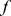, where 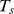 represents the spatial smoothing operator (here corresponding to a two-dimensional Gaussian kernel (24)), can be expressed as



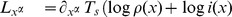


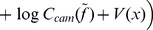
(89)where

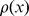
 is a spatially dependent *albedo factor* that reflects *properties of surfaces of objects* in the environment with the implicit understanding that this entity may in general refer to points on different surfaces in the world depending on the viewing direction and thus the image position 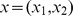,

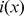
 denotes a spatially dependent *illumination field* with the implicit understanding that the amount of incoming light on different surfaces may be different for different points in the world as mapped to corresponding image coordinates 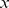,

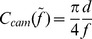
 represents *internal camera parameters* with the ratio 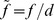 referred to as the *effective* 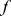 *-number*, where 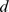 denotes the diameter of the lens and 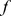 the focal distance,

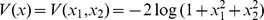
 represents a geometric *natural vignetting* effect corresponding to the factor 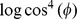 for a planar image plane, with 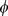 denoting the angle between the viewing direction 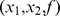 and the surface normal 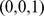 of the image plane. (This vignetting term will disappear for a spherical camera model.)

From the structure of equation (89) we can note that for any non-zero order of differentiation 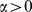, the influence of the internal camera parameters in 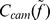 will disappear because of the spatial differentiation with respect to 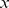, and so will the effects be of any other multiplicative exposure control mechanism. Furthermore, for any multiplicative illumination variation 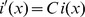, where 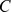 is a scalar constant, the logarithmic luminosity will be transformed as 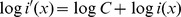, which implies that the dependency on 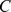 in (87) will disappear after spatial differentiation.

Thus, after a logarithmic transformation of the intensity axis receptive field responses in terms of spatial derivatives are *invariant under multiplicative illumination variations*. For biological vision, such multiplicative exposure control mechanisms correspond to adaptations of the luminosity on the retina by varying the diameter of the pupil as well as adaptations of the light sensitivity of the photoreceptors to the luminosity.

**Figure 16:**
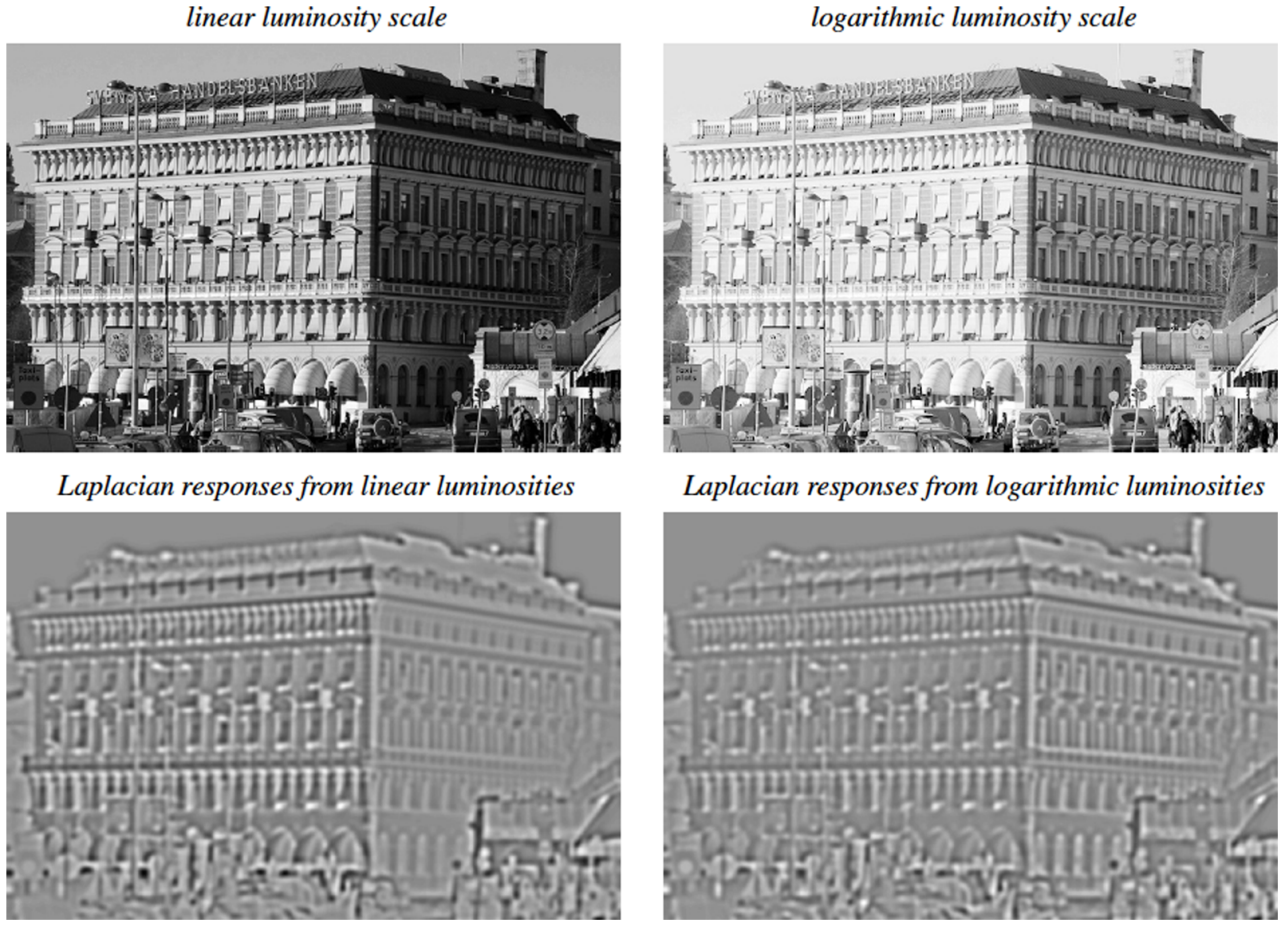
Illustration of the effect of computing Laplacian receptive field responses 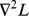 from image intensities defined on (left column) a linear intensity scale 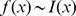 *vs.* (right column) a logarithmic intensity scale 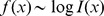 for an image with substantial illumination variations. As can be seen from the figure, the magnitudes of the Laplacian receptive field response are substantially higher in the left sunlit part of the house compared to the right part in the shade if the Laplacian responses are computed from a linear luminosity scale, whereas the difference in amplitude is between the left and the right parts of the house becomes substantially lower if the receptive field responses are computed from a logarithmic intensity scale.

Figure 16 illustrates this effect by showing Laplacian receptive field responses computed with respect to a linear *vs.* a logarithmic luminosity scale for a building for which two different walls are subject to different type of illumination.

Specifically, if the illumination field 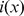 is constant over the support region of the receptive field, the receptive field response will then up to the variations in the natural vignetting 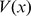 only respond to the spatial variations of the albedo factor 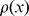, *i.e.*, only to variations in the surface pattern(s) in the world. Hence, *the receptive field responses will have a direct physical interpretation in terms of properties of objects and events in the environment*. This result can be seen as a theoretical explanation of why recognition methods based on receptive field responses work so well in the area of computer vision. More generally, this result could also be seen as a theoretical explanation of how the receptive field responses that are computed in LGN and V1 can constitute the foundation for the visual operations in higher visual areas in biological vision.

Notably, the vignetting effect 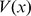 is independent of the image contents 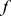 and could therefore be corrected for given sufficient knowledge about the camera. For spatio-temporal receptive fields 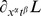 that involve explicit temporal derivatives with 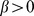, it will furthermore disappear altogether, since the vignetting only depends upon the spatial coordinates.

## Summary and conclusions

**Figure 17:**
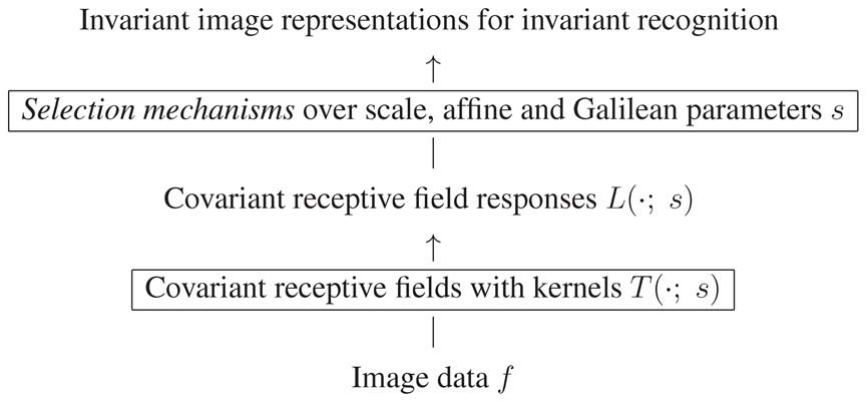
Schematic overview of how the covariance properties of the receptive fields in the proposed receptive field model lead to covariant image measurements, from which truly invariant image representations can then be obtained by complementary selection mechanisms that operate over the parameters 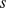 of the receptive fields corresponding to variations over scale, affine image deformations and Galilean motions. For pure scaling transformations, the parameter 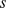 of the receptive fields will be a scalar scale parameter, whereas a covariance matrix 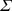 is needed to capture more general affine image deformations. For spatio-temporal image data, an additional temporal scale parameter 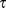 and an additional image velocity parameter 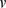 are furthermore needed.

We have described how the shapes of receptive field profiles in the early visual pathway can *be constrained* from structural symmetry properties of the environment, which include the requirement that the receptive field responses should be sufficiently well-behaved (covariant) under basic image transformations. We have also shown how these covariance properties of receptive fields *enable true invariance properties* of visual processes at the systems level, if combined with max-like operations over the output of receptive field families tuned to different filter parameters (see figure 17).

The invariance and covariance properties that we have considered include (i) scaling transformations to handle objects and substructures of different size as well as objects at different distances from the observer, (ii) affine transformations to capture image deformations caused by the perspective mapping under variations of the viewing direction, (iii) Galilean transformations to handle unknown relative motions between objects in the world and the observer and (iv) multiplicative intensity transformations to provide robustness to slowly varying illumination variations as well as invariance to intensity variations caused by multiplicative exposure control mechanisms.

These transformations should be interpreted as *local approximations* of the actual image transformations, which in general can be assumed to be non-linear. Thus, a sufficient requirement for these invariance or covariance properties to be hold in practice and thus enable robust visual recognition from real-world image data, is that these approximations should hold locally within the support region of a given receptive field. Therefore, these theoretical results can be extended to more complex scenes by using different local approximations for receptive fields at different spatial or spatio-temporal points.

The presented theory leads to a computational framework for defining spatial and spatio-temporal receptive fields from visual data with the attractive properties that: (i) the receptive field profiles can be derived *by necessity* from first principles and (ii) it leads to *predictions* about receptive field profiles in good agreement with receptive fields found by cell recordings in biological vision. Specifically, idealized models have been presented for space-time separable receptive fields in the retina and LGN and for non-separable simple cells in V1.

The modelling performed in this article has been performed at a more abstract level of computation than used in many other computational models, and should therefore be applicable to a large variety of neural models provided that their functional properties can be described by appropriate diffusion equations. These results are therefore very general, since they are based on inherent properties of the image formation process, and should therefore have important implications for computational modelling of visual processes based on receptive fields. If one accepts the assumptions underlying the model, these results should therefore have important implications for computational neuroscience, since they hold for any computational model whose functionality is compatible with the assumptions.

Compared to more common approaches of learning receptive field profiles from natural image statistics, the proposed framework makes it possible to derive the shapes of idealized receptive fields without any need for training data. The proposed framework for invariance and covariance properties also adds explanatory value by showing that the families of receptive profiles tuned to different orientations in space and image velocities in space-time that can be observed in biological vision can be *explained* from the requirement that the receptive fields should be covariant under basic image transformations to enable true invariance properties. If the underlying receptive fields would not be covariant, then there would be a systematic bias in the visual operations, corresponding to the amount of mismatch between the backprojected receptive fields.

The theory could also be used as a framework for raising questions concerning invariance properties of biological vision. As a complement to the fundamental covariance properties, we have outlined possible mechanisms for how *true invariance* under scaling transformations, affine transformations and Galilean transformations can be obtained already at the level of receptive field responses. The presented mechanisms are based on two types of major principles; (i) by detecting *extremum values* of appropriately normalized receptive field responses over variations of the filter parameters or (ii) by *normalizing* the receptive field responses with respect to a preferred *reference frame* that is constructed from criteria that are invariant under the corresponding image transformations. These methods have been successfully applied in the area of computer vision and demonstrate how the covariance properties of the proposed receptive field model can be used for defining truly scale invariant, affine invariant and Galilean invariant visual operations already at the level of receptive fields, which can then provide a basis for computational mechanisms for invariant recognition of visual objects and events at the systems level.

We have also described how invariance to local multiplicative illumination transformations and exposure control mechanisms will be automatically obtained for receptive fields in terms of spatial or spatio-temporal derivatives. If we can assume that the illumination varies slowly and can be regarded as constant over the support region of the receptive field, the receptive field response will therefore have a direct physical interpretation as corresponding to variations in the surface structures of objects in environment. Thus, the receptive field responses reflect important *physical properties* of objects and events in the environment to support visual recognition.

It should be emphasized, however, that the model has not been constructed to mimic mammalian vision or the vision system in other species. Instead it is intended as an *idealized* theoretical and computational model to capture inherent properties of basic image transformations that any computational vision model needs to be confronted with.

Concerning limitations of the proposed approach, it should be stressed that a basic requirement for obtaining true invariance with respect to the image transformations according to the proposed invariance mechanisms, is that the vision system has a sufficient number of receptive fields over a *sufficient range* of filter parameters to support invariance over a corresponding range of parameter variations. Notably, such a limitation is consistent with the findings from biological vision that the scale invariant properties of neurons may only hold over finite ranges of scale variations (Ito *et al.* [3]).

It should also be noted that the invariance and covariance properties are only guaranteed to hold if the same local approximation of the image transformation is valid within the entire support region of the receptive field. Thus, complementary mechanisms can be needed to handle, *e.g.*, discontinuities in depth, discontinuities in the illumination field or specularities.

An interesting observation that can be made from the similarities between the receptive field families that have been derived by necessity from the assumptions and the receptive profiles found by cell recordings in biological vision, is that receptive fields in the retina, LGN and V1 of higher mammals are very close to *ideal* in view of the stated structural requirements/symmetry properties. In this sense, biological vision can be seen as having adapted very well to the transformation properties of the outside world and to the transformations that occur when a three-dimensional world is projected to a two-dimensional image domain and being exposed to illumination variations.

Thus, image measurements in terms of receptive fields according to the proposed model can (i) be interpreted as corresponding to image features that are either invariant or covariant with respect to basic geometric transformations and illumination variations and can (ii) serve as a foundation for achieving invariant recognition of visual objects at the system level under variations in viewpoint, retinal size, object motion and illumination.

From a background of the presented theory, we can therefore interpret the receptive fields in V1 as highly dedicated computational units that are very well adapted to enable the computation of invariant image representations at higher levels in the visual hierarchy.

## Discussion

In his recent overview of Bayesian approaches to understanding the brain, Friston [104] writes that “ …we are trying to infer the causes of our sensations based on a generative model of the world” and ‘ …if the brain is making inferences about the causes of its sensations then it must have a model of the causal relationships (connections) among (hidden) states of the world that cause sensory input. It follows that neuronal connections encode (model) causal connections that conspire to produce sensory information”. He furthermore states that an underlying message in several lines of brain research is that the brain is regarded as “optimal in some sense”.

The presented theory can be seen as describing consequences of a similar way of reasoning regarding the development of receptive fields in the earliest stages of visual processing. If the brain is to handle the large natural variability in image data under basic image transformations, such as scaling variations, viewing variations, relative motion or illumination variations, then an optimal strategy may be to adapt to these variabilities by making it possible to respond to image transformations in terms of invariance or covariance properties. If the receptive fields would not be covariant under basic image transformations, then that would imply that some of the variabilities in the information could not be appropriately captured by the vision system, which would affect its performance. By in addition developing invariance properties at higher levels in a visual hierarchy, the brain will be able to deal with natural image transformations in a robust and efficient manner.

Thus, the proposed theory of receptive fields can be seen as describing basic physical constraints under which a learning based method for the development of receptive fields will operate and the solutions to which an optimal adaptive system may converge to. Field [44] as well as Doi and Lewicki [105] have described how €natural images are not random, instead they exhibit statistical regularities€ and have used such statistical regularities for constraining the properties of receptive fields. Receptive field profiles have been derived by statistical methods such as principal component analysis (Olshausen and Field [46]; Rao and Ballard [47]), independent component analysis (Simoncelli and Olshausen [48]; Hyvärinen *et al.* [106]) and sparse coding principles (Lörincz *et al.* [107]). The theory presented in this paper can be seen as a theory at a higher level of abstraction, in terms of basic principles that reflect properties of the environment that in turn determine properties of the image data, without need for explicitly constructing specific statistical models for the image statistics. Specifically, the proposed theory can be used for explaining why the above mentioned statistical models lead to qualitatively similar types of receptive fields as the idealized receptive fields obtained from our theory.

Concerning the closely related issue of how receptive fields are distributed over the visual cortex, Kaschube *et al.* [108] have found that pinwheel density as defined from singularities in the orientation fields of orientation hypercolumns is similar between species that separated evolutionary more than 65 million years ago. By studying structural properties of self organizing systems for idealized neural interaction models, they showed that an overall suppressive nature of non-local long-range interactions is essential for the development of the pinwheel layout observed in carnivores and primates. Thus, the distribution of orientation hypercolumns in the visual cortex can be predicted from internal structural properties of self-organizing neural networks. This paper presents a corresponding theoretical study of how the shapes of receptive field profiles found in the retina, LGN and the striate cortex can be predicted from structural properties of the environment and of how invariance properties can be achieved with a complementary assumption concerning the architecture of complementary selection mechanisms that operate over ensembles of receptive fields.

In terms of computational modelling of vision, the proposed model for covariant receptive fields leading to true invariance properties should require a significantly lower amount of training data compared to approaches that involve explicit learning of receptive fields or compared to computational models that are not based on explicit invariance properties in relation to the image measurements. Specifically, we propose that if the aim is to build a computational vision system that solves specific visual tasks, then a neuro-inspired artificial vision system based on these types of provable invariance properties should allow for more robust handling of natural imaging variations.

### 

#### Note I: Relations to Lie groups

In relation to visual invariances, Miao and Rao [109] have presented an expectation/maximization approach for learning Lie transformation operators and applied this approach for learning affine transformations in the spatial domain. There is a close relationship between derivatives of Lie groups and local linearizations of non-linear transformations as used in our work. Indeed, the directional derivative operator of a multi-parameter scale-space in equation (13) and the connection equations we have previously stated between the different internal representations in a multi-parameter scale-space (Lindeberg [56]) are very closely related to corresponding derivative operators of Lie groups. For the purpose of this presentation, we have, however, avoided explicit use of the Lie group formalism, since local linearizations are sufficient to model the influence of the image transformations on the receptive fields up to first-order of approximation. By this, we have presented a uniform framework for modelling essential effects on receptive fields due to variations in viewing distance, viewing direction, relative motion in relation to the observer and local multiplicative illumination variations, including theoretical necessity results regarding the shapes of the receptive field profiles and a framework for obtaining provable invariance properties under basic image transformations as caused by structural properties of the environment.

#### Note II: Relations between receptive fields in the LGN and V1

In relation to the derived idealized models for linear receptive fields and their close similarities to biological receptive fields measured in the LGN and V1 of higher mammals, a complementary question that one may ask concerns why most receptive fields in the retina and the LGN are rotationally symmetric in the spatial domain and separable over space-time, whereas the more general development of elongated receptive fields over space that permit affine invariance and non-separable space-time tilted spatio-temporal receptive fields over space-time that permit Galilean invariance are primarily developed at later stages essentially with a dominance at first in V1? Could it be because of inherent theoretical reasons, because of the different types of connections from and to other visual areas or because of the way mammalian vision has developed over evolution?

The microstructure of the LGN (Sherman [110]) is different from the microstructure of V1 (Callaway [111]). If we interpret this microstructure as enabling the computations that underlie the formation of the receptive fields, then this in combination with the different connectivities of LGN and V1 to other visual areas indicates that the receptive fields in the LGN *vs.* V1 are likely to have different functions and/or that they have developed at different stages of brain evolution.

Concerning the function of the LGN, experimental evidence have been presented that a major source of information to the LGN comes from top-down connections whose detailed functions remain to be understood (Murphy *et al.* [112], Alitto and Usrey [113], Przybyszewski [114]). Such feed-back mechanisms are beyond the feed-forward model of receptive fields used for deriving the theoretical results in this article except for the general extension to non-linear feedback by steering the conductivities in the diffusion equation that determine the evolution of receptive fields based on local image information that was proposed in connection with equation (72). Such an additional feedback mechanism can also be extended to spatio-temporal image data. If we model the spatial components of the majority of LGN receptive fields by Laplacian of Gaussians, we can interpret these receptive field responses as a bandpass representation of the original image data, with the interesting additional interpretation that the original image data can be reconstructed from a sum of a set of Laplacian of Gaussians at multiple spatial scales
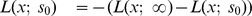


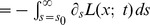


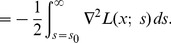
(90)


Temporal derivatives at different temporal scales can in turn be interpreted as a temporal bandpass representation. In the area of image processing, one has learned that it can be easier to merge (fuse) visual information from different sources by operating on a bandpass representation as opposed to the original image data (Burt and Kolczynski [115]). Bandpass representations have also been argued to be optimal from a transmission coding point of view (Zhaoping [116]) by decorrelating the data to be transmitted. Would these be arguments for using a bandpass representation at an early visual processing stage to make it easier to transmit and combine incoming image data from different sources as well as with top-down influence?

Alternatively speaking, if we interpret the LGN as an evolutionary earlier visual processing center based on which higher level visual areas may have developed later (Kaas [117]), could a major reason be that rotationally symmetric and space-time separable receptive fields are evolutionary younger than more refined elongated and space-time tilted receptive fields that allow for affine invariance and Galilean invariance, and which may then have been developed in higher and evolutionary younger parts of the brain?

Concerning the specific use of Laplacian of Gaussian receptive fields in the retina and the LGN, this family of receptive fields spans the subgroup of geometric image transformations over scale. Disregarding translational invariance, which can be seen as implicitly represented by receptive fields distributed over the retina, scale invariance is presumably the type of invariance next at hand that cannot be neglected in a real-world situation, since objects are of different sizes in the world and the perspective mapping leads to scaling transformations due to variations in the distance between objects in the environment and the observer.

A more inherent explanation for the separation between different types of receptive fields between the retina and the LGN *vs.* V1 can also be formulated in terms of computational efficiency and/or timing. The simpler types of receptive fields that are present in the retina and the LGN can be computed faster and/or using a smaller number of neurons, which can then be used as a basis for faster and/or more specific subcortical visual functions, compared the more refined receptive fields in V1 that serve as basis for the more developed higher visual areas in the cortex.

To span the variability of receptive field profiles under variations of all the parameters that determine the orientations and the scales of affine receptive fields as well as a sufficiently rich set of motion directions for velocity-adapted receptive fields to be able to achieve invariance over wide ranges of variations in scale, viewing directions and relative motions, the full group of such covariant receptive fields in V1 will therefore require a significantly larger number of neurons to represent the receptive fields that correspond to all possible parameter setting in comparison with the much lower variability in the subgroup of image transformations over scale that can be captured by rotationally symmetric and space-time separable LGN cells.

Notably the LGN of a higher mammal may contain the order of 1 to 4 million neurons which is comparable to the number of output axons from the retina, whereas V1 may contain the order of 100 to 1000 million neurons (Stevens [118]). Therefore, for lower level visual functions that may not require the more developed invariance properties possible from V1 output, requirements of efficiency may call for such functions to be implemented in terms of simpler receptive fields that are only able to represent a lower-dimensional subgroup within the more general group of natural image transformations. For certain specific tasks such as signalling new events for attention, handling eye movements or reacting as fast as possible to possible threats, rotationally symmetric and space-time separable receptive fields may be sufficient and thus more effective, whereas more general visual tasks related to *e.g.* invariant recognition of objects and events under natural image transformation may require the full machinery made possible by the large variabilities of receptive field profiles in V1 as induced by covariance properties under natural image transformations.

From arguments of this type, it seems possible that one may extend the theoretical model proposed in this article by complementary notions and possibly further experimental evidence to address these issues concerning the internal structure of the early visual areas in further detail.
